# Between-Habitat Variation of Benthic Cover, Reef Fish Assemblage and Feeding Pressure on the Benthos at the Only Atoll in South Atlantic: Rocas Atoll, NE Brazil

**DOI:** 10.1371/journal.pone.0127176

**Published:** 2015-06-10

**Authors:** G. O. Longo, R. A. Morais, C. D. L. Martins, T. C. Mendes, A. W. Aued, D. V. Cândido, J. C. de Oliveira, L. T. Nunes, L. Fontoura, M. N. Sissini, M. M. Teschima, M. B. Silva, F. Ramlov, L. P. Gouvea, C. E. L. Ferreira, B. Segal, P. A. Horta, S. R. Floeter

**Affiliations:** 1 Programa de Pós-Graduação em Ecologia, Universidade Federal de Santa Catarina, Florianópolis, Brazil; 2 Laboratório de Biogeografia e Macroecologia Marinha, Departamento de Ecologia e Zoologia, Universidade Federal de Santa Catarina, Florianópolis, Brazil; 3 Laboratório de Ficologia, Departamento de Botânica, Universidade Federal de Santa Catarina, Florianópolis, Brazil; 4 Laboratório de Ecologia e Conservação de Ambientes Recifais, Universidade Federal Fluminense, Niterói, Brazil; 5 Laboratório de Ecologia de Ambientes Recifais, Departamento de Ecologia e Zoologia, Universidade Federal de Santa Catarina, Florianópolis, Brazil; 6 Laboratório de Crustáceos e Plâncton, Departamento de Ecologia e Zoologia, Universidade Federal de Santa Catarina, Florianópolis, Brazil; 7 Instituto Chico Mendes de Conservação da Biodiversidade (ICMBio), Reserva Biológica do Atol das Rocas, Natal, Brazil; 8 Instituto Coral Vivo, Arraial d'Ajuda, Porto Seguro, Brazil; University of California Santa Cruz, UNITED STATES

## Abstract

The Southwestern Atlantic harbors unique and relatively understudied reef systems, including the only atoll in South Atlantic: Rocas atoll. Located 230 km off the NE Brazilian coast, Rocas is formed by coralline red algae and vermetid mollusks, and is potentially one of the most “pristine” areas in Southwestern Atlantic. We provide the first comprehensive and integrative description of the fish and benthic communities inhabiting different shallow reef habitats of Rocas. We studied two contrasting tide pool habitats: open pools, which communicate with the open ocean even during low tides, thus more exposed to wave action; and closed pools, which remain isolated during low tide and are comparatively less exposed. Reef fish assemblages, benthic cover, algal turfs and fish feeding pressure on the benthos remarkably varied between open and closed pools. The planktivore *Thalassoma noronhanum* was the most abundant fish species in both habitats. In terms of biomass, the lemon shark *Negaprion brevirostris* and the omnivore *Melichtys niger* were dominant in open pools, while herbivorous fishes (mainly *Acanthurus* spp.) prevailed in closed pools. Overall benthic cover was dominated by algal turfs, composed of articulated calcareous algae in open pools and non-calcified algae in closed pools. Feeding pressure was dominated by acanthurids and was 10-fold lower in open pools than in closed pools. Besides different wave exposure conditions, such pattern could also be related to the presence of sharks in open pools, prompting herbivorous fish to feed more in closed pools. This might indirectly affect the structure of reef fish assemblages and benthic communities. The macroalgae *Digenea simplex*, which is uncommon in closed pools and abundant in the reef flat, was highly preferred in herbivory assays, indicating that herbivory by fishes might be shaping this distribution pattern. The variations in benthic and reef fish communities, and feeding pressure on the benthos between open and closed pools suggest that the dynamics in open pools is mostly driven by physical factors and the tolerance of organisms to harsh conditions, while in closed pools direct and indirect effects of species interactions also play an important role. Understanding the mechanisms shaping biological communities and how they scale-up to ecosystem functioning is particularly important on isolated near-pristine systems where natural processes can still be studied under limited human impact.

## Introduction

Reef ecosystems around the globe have suffered from a variety of anthropogenic activities including habitat degradation, overfishing, coastal pollution, introduction of invasive species and global warming, that affected habitat complexity, physical characteristics (*e*.*g*. temperature, pH, turbidity), species diversity, biotic interactions and the ecosystem processes they mediate [1–3]. The combination of species interactions and abiotic conditions shape the complexity of reef systems, which highlights the need to understand the relative contribution of these components to ecosystem structure and functioning [4–5]. Physical factors, such as wave exposure and tidal currents, have been recognized as one of the main forces regulating reef structure and dynamics [6–7]. For instance, the diversity and cover of hard corals can be negatively related to wave exposure, as it can result in physical damage to less robust branching corals. On the other hand, turf algae cover can prevail in exposed habitats because of its tolerance to disturbances and ability to colonize newly available substrate [8–9]. In dynamic systems, such as atolls, the tidal regime is particularly important in determining current strength, nutrient availability and particulate matter, hence influencing benthic communities [7, 10]. Likewise, reef fish communities respond to wave-induced water motion according to species’ swimming abilities [11–12]. Exposed areas can favor planktivores and piscivores, while site-attached species with limited swimming capability, such as territorial pomacentrids, tend to live closely associated with the reef [13]. These physical factors can also influence fish feeding behavior [14]. At the Great Barrier Reef, for instance, reef fish herbivory varied among habitats and exposure conditions, with higher rates of macroalgae removal in more exposed sites [15], potentially impacting the structure of benthic communities.

The effect of herbivory on reef structure and dynamics is largely recognized as a critical ecological process in coral reefs [2, 16–19]. A meta-analysis exploring the relative importance of herbivory (top-down force) and nutrient supply (bottom-up force) in structuring benthic communities found that herbivory can exert a stronger effect on tropical macroalgae and seagrass than nutrient supply [20]. When herbivorous fishes were excluded from reef areas both in the Caribbean and the Great Barrier Reef, macroalgae rapidly outgrew other benthic organisms, revealing a critical top-down control [19, 21–22]. However, the ability of herbivores to control macroalgae also depends on a combination between algal traits (e.g. defenses, nutritional value) and herbivore diversity, reflected, for instance, in their tolerance to anti-herbivore defenses and feeding preferences [23]. Thus, the relative contribution of ecological processes and physical factors in structuring reef communities may have context-dependent effects, varying within and between-habitats [4]. Understanding these factors is critical for informed conservation strategies, for example by protecting critical ecological processes and habitats with different abiotic conditions [2, 10].

The South Atlantic Ocean harbors unique reef systems with different characteristics and dynamics when compared to the Indo-Pacific and Caribbean, as a result of different historical and biogeographical factors (e.g. isolation, biogeographic barriers, reef type, geomorphological features [24–28]). A number of isolated oceanic islands, most of volcanic origin, are scattered along this region and four of these are closer to the Brazilian mainland: Saint Peter and Saint Paul Archipelago, Fernando de Noronha Archipelago, Rocas atoll and Trindade and Martin Vaz Island Complex [25]. Scientific knowledge on marine communities in these islands have improved in the last two decades, especially regarding reef fish assemblages and benthic communities (e.g. [27–38]). These studies have reinforced the notion that these systems are unique and differ from other tropical reefs because of the low species richness and negligible contribution of hard corals to the reef structure.

Among these oceanic reef systems is the only atoll formation in the South Atlantic Ocean: Rocas atoll, 230 km off the northeastern coast of Brazil at the state of Rio Grande do Norte [27]. Unlike most atolls in the world, Rocas is not predominantly constructed by corals, but by coralline algae, vermetid gastropods and encrusting foraminiferans [28]. Rocas is also smaller than most atolls, comprising an area of 5.5 km^2^ [34], in comparison to others such as Palmyra Atoll in the Pacific with ca. 52 km^2^ [9] and Glover’s Reef in the Caribbean with ca. 260 km^2^ [10]. Despite these differences, Rocas has equivalent habitats such as: a shallow lagoon, small sandy islands, algal crest and different reef zones [39, 29]. Additionally, it was the first Brazilian marine protected area, established in 1978, and one of the first no-entry marine reserves in the world [40], being potentially the most effective marine protected area in Brazil. Rocas is also a very dynamic ecosystem prone to the arrival and establishment of new species both through natural or human-mediated processes, with potential consequences to the ecosystem function that are still unknown in terms of magnitude and duration [35].

Despite the uniqueness of Rocas atoll and some staggered efforts to describe its reef structure, fish assemblages, benthic communities and herbivory patterns (e.g., [27–29, 32, 34]), an integrated approach is still missing. Here we provide the first comprehensive and integrative description of patterns of reef communities and fish trophic interactions on the benthos in this unique reef system. Particularly, we describe and compare habitats with different wave exposure regarding: (1) the structure of reef fish assemblages; (2) benthic community; (3) composition, nutritional value and associated cryptofauna of algal turfs; (4) fish feeding pressure on the benthos and herbivory. We expected that: (1) fish species with higher mobility (*e*.*g*., sharks and jacks) would be more common in more exposed habitats; (2) algal turfs would be more abundant in more exposed habitats, however with a lower abundance of associated cryptofauna and lower nutritional value; and (3) a higher feeding pressure would be expected in less exposed habitats, mostly by herbivorous fishes. Alternatively, because Rocas atoll is a very dynamic and isolated system, species with the ability to inhabit such area could be more versatile and thus less affected by local habitat variation. If this was true, then we should expect similar reef communities across different habitats.

## Materials and Methods

### Study Area

**Fig 1:**
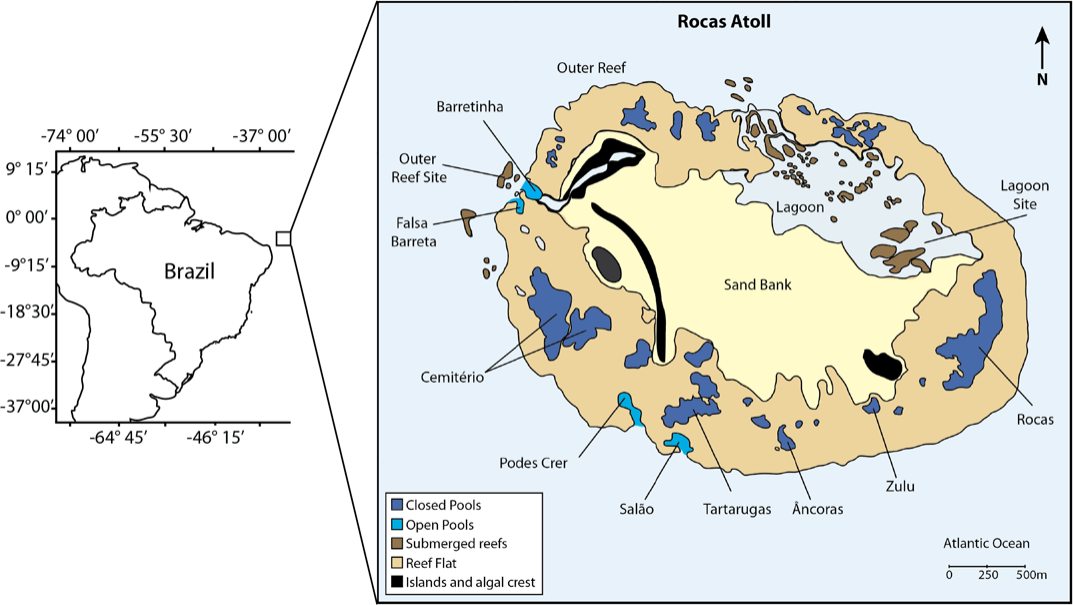
Studied areas at Rocas atoll, NE Brazil, indicating the different habitats. The large scale map was plotted with the R software using the 'maptools' package [41] based on data provided by the GEOphysical DAta System—Next Generation (GEODAS-NG) of the NOAA (https://www.ngdc.noaa.gov/mgg/geodas/geodas.html). The illustration of the atoll was adapted from [39] but is not identical.

Rocas atoll is located in the South Atlantic Ocean laying approximately 230 km off the NE coast of Brazil (03°50’S, 33°49’W). Rocas is the only atoll formation in the South Atlantic and is part of a seamount chain in the E-W direction known as the Fracture Zone of Fernando de Noronha [27]. The atoll is subject to an intense wave action in comparison to coastal systems, with predominant winds from S and SE, that lead to an intense wave action in this side of the atoll; although the leeward side can also be occasionally affected by large wave surges [28]. Sea surface temperature in the atoll rim varies between 27°–29°C, while in shallow habitats inside the atoll it may vary between 24°–36°C [34]. The tides range from 0–3.8 m in a semi-diurnal and mesotidal regime [28], resulting in a half-daily cycle of almost complete submersion during high tide (only the sandy islands remain emerged) and almost complete emersion during low tide. The available reef area in its internal portion during the low tide, when tidal currents have ceased, can be distinguished in three main habitats: the shallow permanent lagoon, open and closed pools. Open pools communicate with the exterior of the atoll even during low tides and are more exposed to wave action than closed pools, which remain completely isolated from the exterior area of the atoll during low tide (Fig 1). This tidal dynamics results in strong currents when the atoll is either filling or draining and during high tides [28], reason why diving inside the atoll is concentrated during low tide. Established as a marine reserve in 1978, only in 1991 constant and effective enforcement was implemented through the establishment of a permanent monitoring station at the atoll. Rocas figures as an important study area and natural laboratory because: (1) it is an unique atoll formation and the only one in the South Atlantic; (2) it offers a great variety of habitats with different conditions and under the influence of tidal dynamics; (3) it is probably the most effective marine reserve and most similar to a pristine reef in the Tropical Southwestern Atlantic (SISBIOTA–Mar *unpublished data—* www.sisbiota.ufsc.br).

### Ethical consideration

This study was conducted in accordance with all ethical guidelines and Brazilian legislation, including authorization to assess the Rocas atoll Reserve, to observe, video, photograph and collect the studied organisms, under the permit # 29953–1 (ICMBio/ MMA—Brazilian Ministry of Environment). This permit evaluated and approved the collection and sacrifice methods of fish which also followed the resolution of the Federal Biology Council (CFBio; Resolução N°301, 8 de dezembro de 2012). In order to ameliorate suffering of collected fish, individuals were laid on ice in a thermal box right after collection in accordance with ICMBio authorized procedures. The collections did not involve endangered or protected species.

### Data collection

Fieldwork was conducted during the austral summer (January to February 2012), always in low tide conditions (except for the outer reef sampling that occurred during high tide) and between 09:00–16:00 h. Four different habitats were studied, with depth varying from 2 to 10 m: open pools, closed pools, the lagoon and one outer reef site (see S1 Table). Water temperature during fieldwork varied between 27^o^–29^o^C.

#### Reef fish assemblages

The structure of fish assemblages was assessed through underwater visual censuses during low tide in 10 sites inside the atoll (five closed pools, four open pools and the lagoon) and in one site at the outer reef during high tide. Visual censuses consisted of belt transects in which a diver identified, counted and estimated the total length of fish species inside an area of 40 m² (20 x 2 m, [13]). The same diver returned searching for small, cryptic and hidden species. Each fish was assigned to a functional group following the literature (see S3 Table for the categories [42–44]). Fish biomass was estimated using length–weight relationships available in the literature (e.g. [45]). A total of 153 visual censuses were performed along the four studied habitats and the number of transects in each habitat varied from 5 to 25, depending on the available area (S1 Table).

#### Reef fish feeding pressure on the benthos

Reef fish feeding pressure on the benthos was evaluated through remote video recordings of 2 m^2^ reef areas, demarcated with a 2 m transect tape, that was removed within the first minute of the video [43–44]. A total of 85 reef areas were sampled, 40 in open and 45 in closed pools (see S1 Table). Each area was recorded for 15 min and the central 10 min of each video analyzed. Every fish recorded feeding on the benthos was identified, assigned into a functional group, had its total length estimated based on the transect tape initially deployed, and its bites on reef substratum were counted during the observational period [44]. Feeding pressure was determined by the product of number of bites taken and body mass (kg) of each fish, to account for potential body size variation in bite impact [15, 44]. Individual body mass was obtained using the same procedure described for estimating fish biomass in transects. Reef fishes were assigned to the same functional groups used for fish assemblages, from which only six were recorded feeding on the benthos: scrapers, fine browsers, territorial herbivores, sessile invertebrate feeders, mobile invertebrate feeders and omnivores. Thus, fish feeding pressure on the benthos was evaluated from the perspective of several functional groups within different trophic categories and accounted for body size variation, per unit of time and area [(Bites x kg) / (2 m² x 10 min)] [44].

#### Benthic cover

Inside each of the recorded areas, benthic cover was estimated using a set of five 25 x 25 cm photoquadrats. Each photograph was analyzed with the software Coral Point Count with Excel extensions [46]. Fifty points were randomly positioned over each image and the organism below each point was identified into morpho-functional groups, by species or genus level [47–50]. Sponges, ascidians and cyanobacteria were kept as broad groups due to limitations in identifying these groups in the photoquadrats [50] and algae were identified to the lowest taxonomic level as possible. Algal assemblages were classified as turfs when they formed thick mats, with a low lying layer of tightly packed algae less than 2 cm high (*sensu* [51–53]) and divided in calcareous or non-calcified turfs according to the dominant algae [54].

#### Algal turfs

In this study, the term *algal turfs* refers to the algal component of the complex epilithic algal matrix, which include sediment, detritus and cryptofauna hosted by the algal mats [52–53]. In two closed (Tartarugas and Rocas) and two open pools (Falsa Barreta and Podes Crer), 10 x 10 cm quadrats were haphazardly positioned inside the recorded areas and algal turfs within these quadrats were scraped and collected until the bare reef was apparent. A total of twenty quadrats were collected, equally distributed among two closed and two open pools (*i*.*e*. five samples per pool; see S1 Table). The samples were frozen right after collection, defrosted in the lab and washed with ammonium formiate to remove salts and sand from macroalgal thalli prior to identification. Identified species were dried separately at 38°C (± 2°C) for 24 hours to determine the dry weight as a measure of biomass. Subsequently, all dried species within a sample were combined, powdered in liquid nitrogen and aliquots were separated for nutritional analyzes. The cryptofauna specimens were also separated and further identified to the lowest taxonomic level possible. This sampling method might be underestimating the cryptofauna’s abundance, therefore the interpretation of such data in comparison to other studies with more specific methods should be made cautiously and on a relative basis.

#### Herbivory Assays

Multiple-choice herbivory assays were conducted to quantify algal removal and selectivity by herbivores [23, 55–56]. Seven macroalgae species (*Caulerpa verticillata*, *Canistrocarpus cervicornis*, *Dictyopteris jolyana*, *Dictyopteris plagiogramma*, *Digenea simplex*, *Padina gymnospora* and *Sargassum* sp.) were collected from open pools and from the reef flat and transplanted to a closed pool (Tartarugas). These algae were chosen because of their relatively high abundance in open pools and on the reef flat, contrasting to their low abundance in closed pools, with herbivory being suggested as the main driver of such pattern (see [32]). Algae were collected in the same day of the experiment, placed in a mesh bag and rotated ten times to remove the excess of water before being weighted. Algae were then attached to a 1 m length rope in randomized species orders, distant at least 10 cm from each other. All the ropes were transported in buckets to the experiment site and one of them was placed in a cage of 2 cm mesh size to control for biomass loss due to hydrodynamics and handling procedures. Ropes and controls were placed over the sand bottom adjacent to the reef. An underwater video camera was positioned in front of each rope, approximately 1 to 2 m from the assay, to record the feeding activity of herbivorous fishes over the transplanted algae and surrounding substrate. Because of the tidal conditions, the assays were conducted for two hours and algae were re-weighted right after this period using the same procedure prior to the trials. The videos were analyzed for the entire period or until a reduction of 70–80% of one of the algae [23], which occurred in most videos within 37 minutes on average (9 from 11). The proportion of consumed algae was calculated through the formula: [Wr_i_ x (Wc_F_)/Wc_i_] / Wr_F,_ where Wr_i_ and Wr_F_ are, respectively, the initial and final algae biomasses in the trial rope, and Wc_i_ and Wc_F_ are the initial and final algae biomasses in the control rope, respectively [23]. A total of 13 trials were conducted within 3 days, from which 11 were coupled with video recording.

#### Algal nutritional quality

Total protein, soluble sugars and starch contents from algal turf samples and species used in the herbivory assays were taken from dried and milled aliquots. Lipid content was determined only for species used in the herbivory assays, through the gravimetric procedure developed by [57] and modified from [58]. The extraction of total proteins was performed according to [59]. An aliquot of 50 mg was extracted with 2 ml of sodium hydroxide solution (NaOH) 0.1 mol/L and centrifuged twice at 3000 rpm for 5 min. The supernatants of both extractions were pooled and total soluble protein contents were determined according to [60], using the reagent Coomassie brilliant blue G-250 and BSA as standard. The extraction of total soluble sugars was performed according to [61]. An aliquot of 50 mg was extracted with 2 ml of methanol:chloroform:water (MCW; 12:5:3) and centrifuged at 3000 rpm for 5 min. The supernatant was recovered and the pellet was re-extracted using 2 ml MCW. One part of chloroform and 1.5 part of water were added to each four parts of supernatant, followed by centrifuging at 3000 rpm for 5 min, from which two phases were obtained. The upper aqueous phase was collected and dosage was estimated using anthrone 0.2% [62]. Starch extraction was performed according to [63]. Pellets used in total soluble sugar extraction were ground with perchloric acid (HClO4) 30% (v/v) and centrifuged at 3000 rpm for 5 min. The supernatant was collected and the precipitate was extracted again as specified above. The extract was also centrifuged and the supernatants of both extractions were pooled and analyzed according to [62], using the reagent anthrone 0.2% (w/v). Sugar and starch concentration were calculated using D-glucose as standard.

#### Diet of herbivorous fish

Individuals of *Acanthurus chirurgus* (n = 14) and *Acanthurus coeruleus* (n = 12) were collected with hand spears in the closed pools of Rocas, Âncoras and Tartarugas in May 2013. Fish were collected in the low tide and during the afternoon to assure they had full guts, since this is the expected period of higher feeding activity for most nominally herbivorous fishes [64]. After collection, all individuals were measured to the nearest millimeter (total length) and had their stomach removed and preserved in formalin. In the laboratory, the whole stomach contents of each individual was spread in a Petry dish over a graph paper with 50 random points. Items on top of each of these points were identified using a stereoscopic microscope to the lowest taxonomic category when possible [65].

### Data Analysis

#### Multivariate approach

Differences in the structure of reef fish assemblages (response variable) between the four habitat types (grouping variables; open and closed pools, lagoon and outer reef) were assessed using an analysis of similarity (ANOSIM [66]) and a multidimensional scaling (MDS [66]; square-root transformed biomass data; Bray-Curtis similarity). An ANOSIM was also used to assess differences in the composition of benthic cover (response variables; arcsin transformed; Euclidean distance) between open and closed pools (grouping variables). The relation between the grouping of samples and benthic categories (response variables) between closed and open pools (grouping variables) was assessed through a principal components analysis (PCA; arcsin transformed; Euclidean distance). Differences in both the composition of algal turfs (dry weight) and associated cryptofauna (density) between closed and open pools (grouping variables) were evaluated using ANOSIM (square-root transformed data; Bray-curtis similarity). Appropriate data transformations and association coefficients were chosen based on the nature of the data (i.e., continuous, proportion) following [67].

#### Univariate approach

Independent t-tests on square-root transformed data were used to compare open and closed pools (grouping variables) in terms of (response variables): (1) mean biomass of *Acanthurus chirurgus* and *A*. *coeruleus*; (2) percent cover of the main benthic groups; (3) mean total dry weight of algal turfs, total density of cryptofauna and mean density of each cryptofauna group; (4) nutritional quality of algal turfs (total protein, soluble sugars and starch); and (5) mean total feeding pressure. Differences in the concentration of nutritional components (sugar, starch, protein and lipid) of algae used in the herbivory assay (grouping variables) were independently assessed using a one-way analysis of variance (ANOVA) on square-root transformed data to meet parametric assumptions. The relation between the mean total dry weight of algal turf (independent variable) and the mean total density of cryptofauna (dependent variable) was investigated using a linear regression. In the herbivory assays, differences in the mean proportion of consumed biomass (response variable) among algae species (grouping variables) were evaluated through a Friedman’s test followed by Friedman *a posteriori* multiple comparison tests using square-root transformed data. The same approach was used to compare the mean number of bites (response variable) between algae species (grouping variable) for the two herbivore species. A paired t-test on square-root transformed data was used to assess differences in the proportion of bites taken by each fish species (response variables) on each algae species (grouping variables). Differences in the relative abundance (response variable) of dietary items (grouping variables) of herbivorous fishes were evaluated through a Friedman’s test followed by Friedman *a posteriori* multiple comparison tests.

#### Permutational approach

The selectivity patterns were investigated using the Strauss’ Linear Selection Index (L):L = r_i_—p_i_, where r_i_ is the number of bites taken from alga *I*, as a percentage of the total number of bites from all algae in each assay, and p_i_ is the mass of algae *I* in relation to the total algal mass presented at the beginning of each assay [55, 68]. Thus, different values of selectivity indices were obtained for each algae and each of the two herbivorous species and averaged over all the assays. A 95% confidence interval (CI) was generated for each averaged index through 1,000 iterations of the observed values. CI intervals higher than 0 indicate selection, lower than 0 indicate avoidance and intervals that include 0 indicate that the selection of the algae did not differ significantly from random [55]. Data from herbivory assays did not vary significantly across days and were grouped in all these analysis (S2 Table).

## Results

### Reef fish assemblages

**Fig 2:**
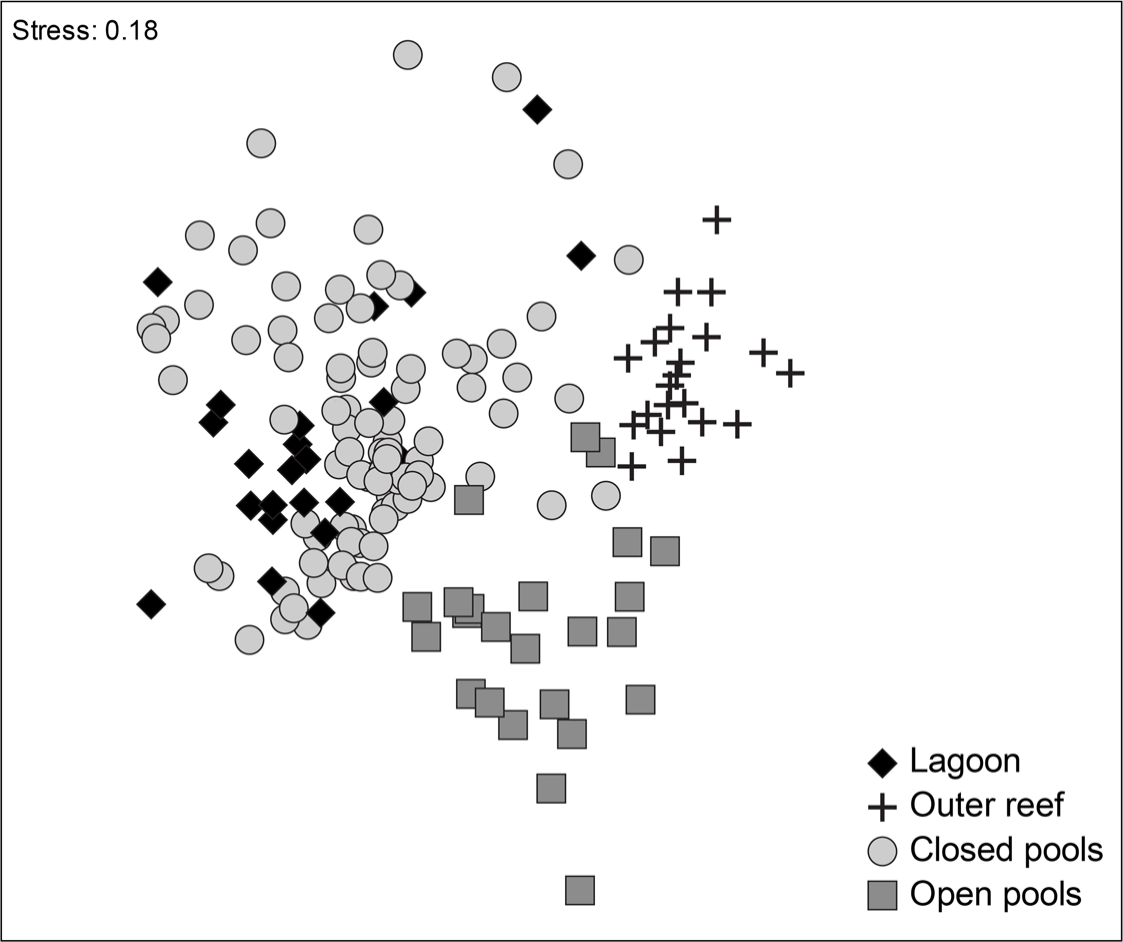
Multidimensional Scaling (MDS) of the composition of reef fish assemblages among different habitats based on a Bray-Curtis similarity matrix.

A total of 53 fish species distributed in 28 families and 10 functional groups were recorded across four studied habitats (S3 Table; closed pools, open pools, the lagoon and the outer reef). The structure of fish assemblages differed between open and closed pools (ANOSIM_Open-Closed_; R = 0.50, p = 0.001), and the outer reef site (ANOSIM_Open-Outer reef_; R = 0.78, p = 0.001; ANOSIM_Closed-Outer reef_; R = 0.70, p = 0.001; Fig 2). The lagoon assemblage differed from the open pools (ANOSIM_Lagoon-Open_; R = 0.68, p = 0.001) and the outer reef (ANOSIM_Lagoon-Outer reef_; R = 0.79, p = 0.001), but not from closed pools (ANOSIM_Lagoon-Closed_; R = 0.10, p = 0.07), and thus was treated as a closed pool in further analysis of reef fish assemblage structure.

**Fig 3:**
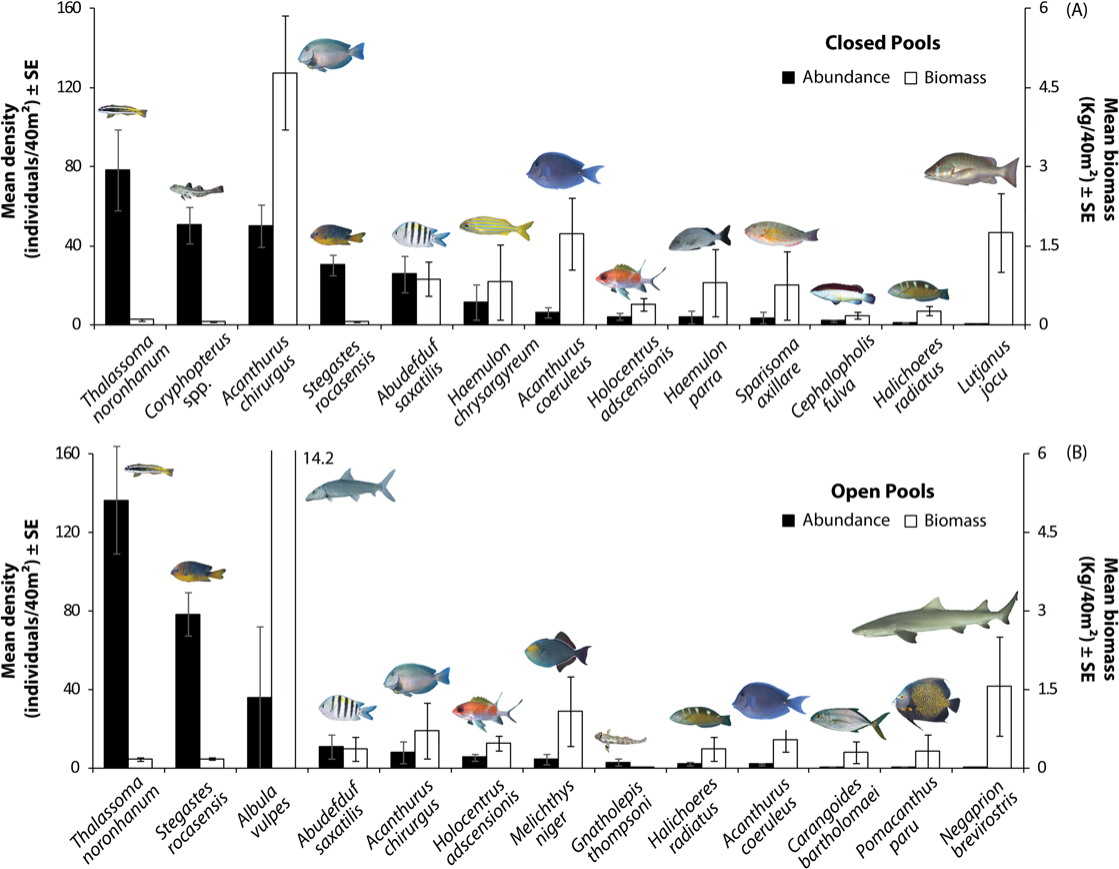
Density and biomass of reef fishes in open and closed pools at Rocas atoll. Displayed species were chosen based on a ranking combining density and biomass. Error bars represent standard errors.

Diurnal planktivores, omnivores, territorial herbivores and scrapers were the most abundant fish functional groups, while scrapers, macrocarnivores and mobile invertebrate feeders comprised most of the fish biomass (S1 Fig). Macrocarnivores were represented especially by apex predators, such as the dog snapper, *Lutjanus jocu*, the lemon shark, *Negaprion brevirostris*, and the nurse shark, *Ginglymostoma cirratum*. *Thalassoma noronhanum* was the most abundant species in both closed and open pools, but *Stegastes rocasensis* was also abundant in both habitats (Fig 3). *Coryphopterus* spp. and *Acanthurus chirurgus* were particularly abundant in closed pools while *Albula vulpes* occurred only in one of the open pools (Barretinha), in large schools associated to sandy patches (Fig 3), comprising a high biomass. Since these schools were ephemeral and spatially localized, they were excluded from the ordination analysis. Apart from this species, biomass in open pools was composed mainly by the shark *N*. *brevirostris*, followed by *Melichthys niger*, *A*. *chirurgus* and *A*. *coeruleus*. Schools of *A*. *chirurgus* comprised the greatest biomass in closed pools (Fig 3), with *L*. *jocu*, and *A*. *coeruleus* also contributing considerably. The biomass of the two most abundant herbivorous fishes in the atoll, *A*. *chirurgus* and *A*. *coeruleus*, were respectively five and three times higher in closed pools in comparison to open pools (t-test for *A*. *chirurgus*: t = 7.02, p<0.001; t-test for *A*. *coeruleus*: t = 2.16, p<0.05; S2 Fig).

### Benthic cover

**Fig 4:**
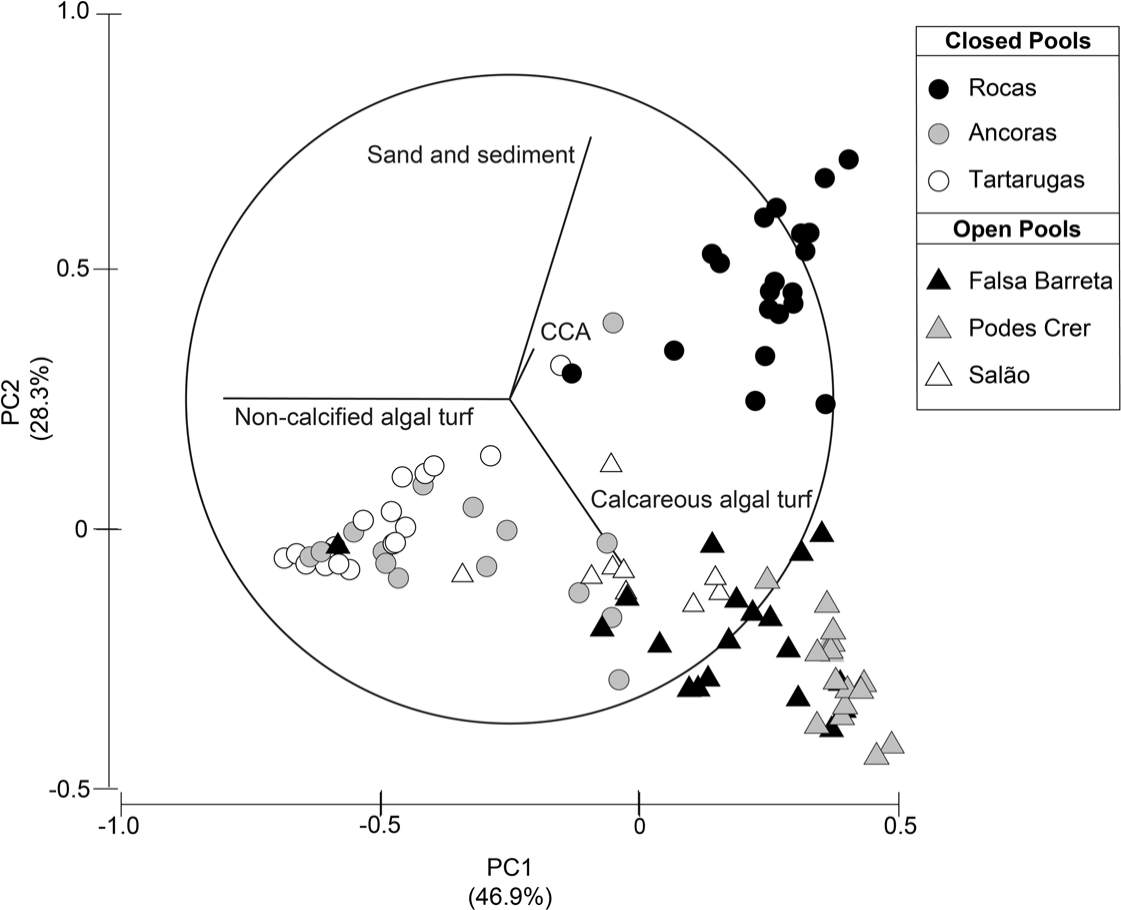
Principal component analysis (PCA) on the composition of benthic cover of closed and open pools. CCA—Crustose coralline algae.

**Fig 5:**
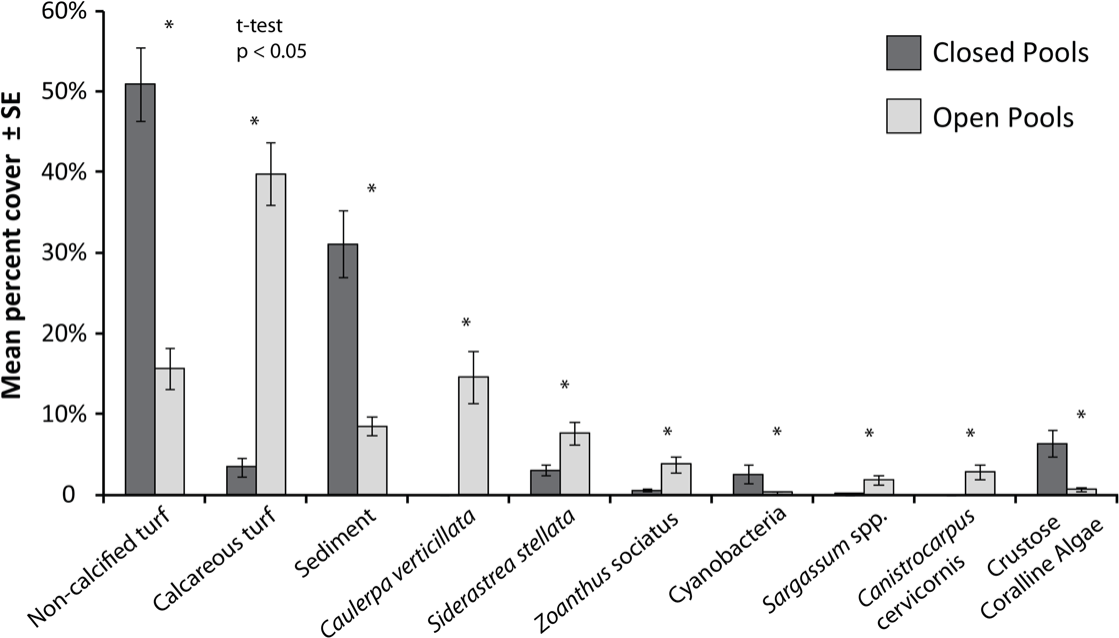
Mean percent cover of the main benthic groups (i.e. those that pooled comprise between 80 and 100% of total cover). (*) Indicates significant differences between closed and open pools (t-test, p<0.05; S5 Table). Error bars represent the standard errors.

The benthic community was characterized by 37 functional and taxonomic groups and presented different physiognomy between open and closed pools (S4 Table; ANOSIM_Open-Closed_; R = 0.53, p<0.001; Fig 4). Differences in algal turfs (dominated by non-calcified or articulated calcareous algae, in closed and open pools respectively) and the cover of sediment determined the grouping of samples between closed and open pools, and the variability within these categories. While samples from open pools were grouped by calcareous algal turfs, irrespective of pool identity, samples from closed pools were separated in those characterized by non-calcified algal turfs (pertaining to Âncoras and Tartarugas) and Rocas pool that presented a large amount of sediment covering the reefs and often the algal turfs (Fig 4). The most abundant group in closed pools was the non-calcified algal turfs (51%), followed by sediment (31%) and crustose coralline algae (6%). In open pools, algal turf dominated by articulated calcareous algae was the most abundant group (33%), followed by the alga *Caulerpa verticillata* (15%) and non-calcified turf (14%). The percent cover of all main benthic groups (i.e. those that pooled comprised between 80 and 100% of the cover) differed between closed and open pools, with closed pools presenting three times more sediment than open pools (Fig 5; S5 Table). Hard corals were mainly represented by *Siderastrea stellata*, with a significantly higher cover in open pools (8%) in comparison to closed pools (3%; Fig 5). The corals *Favia gravida*, *Mussismilia hispida* and *Porites astreoides* were also recorded in samples, but represented less than 1% of benthic cover. Although it was not recorded in the photoquadrats because of its low abundance, the coral *Madracis decactis* was also observed during fieldwork.

### Algal turfs, associated cryptofauna and nutritional traits

**Fig 6:**
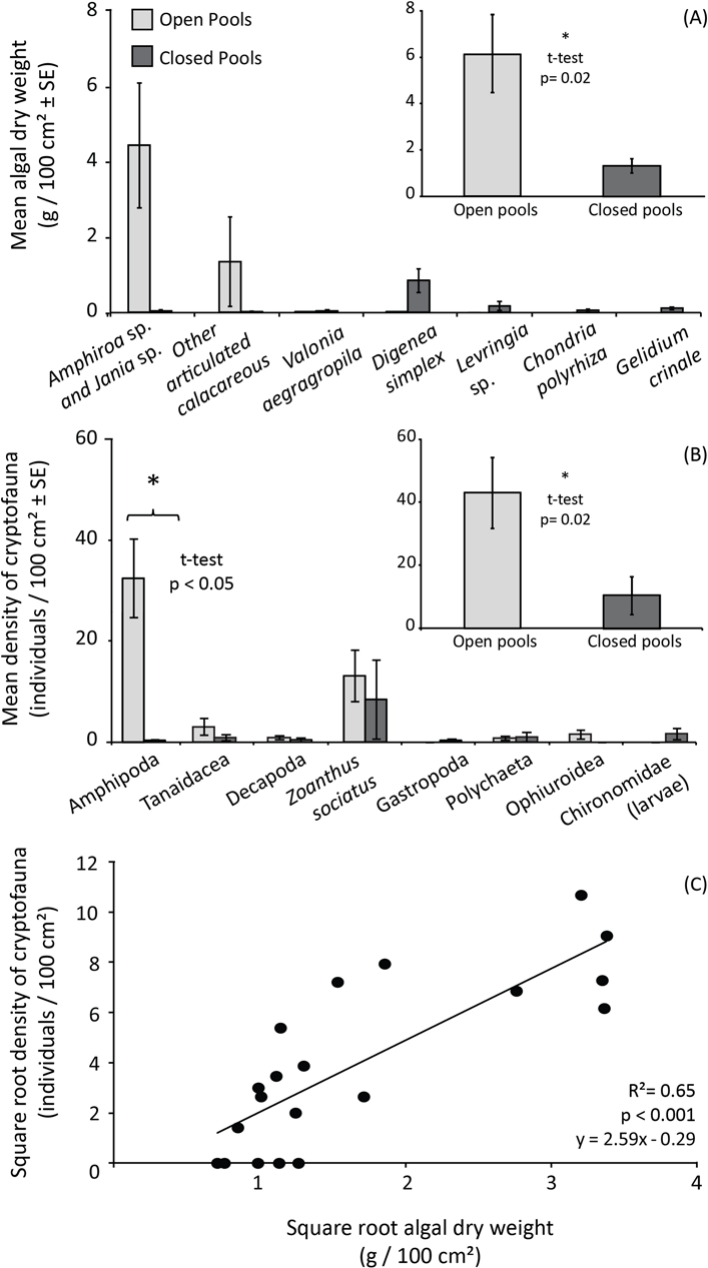
Algal turf species composition and density of associated cryptofauna in open and closed pools. (A) Mean biomass of the main turf-forming macroalgae; (B) mean density of cryptofauna associated to algal turfs; (C) correlation between cryptofauna density and algal turf biomass. The presented algal species combined account for 90% of total biomass in these habitats. Error bars represent the standard errors.

Algal turf assemblages were composed by 47 infrageneric macroalgae taxa, with 9 species and 6 genera that are new records to Rocas atoll (S6 Table). Total algal biomass was three times higher in open pools than in closed pools (t-test; t = 2.66, p = 0.016). Algal turf composition also varied between closed and open pools (ANOSIM_Closed-Open_; R = 0.48, p<0.002) based on their biomass (Fig 6A). Rhodophyta was the most representative group both in terms of species richness and biomass (e.g. *Amphiroa* sp., *Jania* sp., *Digenea simplex*, *Gelidium crinale*). While algal turfs in closed pools were predominantly composed by small-cropped thallus of the red alga *D*. *simplex* and other non-calcified algae, articulated calcareous algae (*e*.*g*. *Jania* sp. and *Amphiroa* sp.) were the major component in open pools (Fig 6A). Similarly, the mean density of cryptofauna on algal turfs from open pools was roughly five times higher than on algal turfs from closed pools (t-test; t = 2.49, p<0.05; Fig 6B). Invertebrates from five different phyla were recorded and identified to different taxonomic levels (S7 Table), depending on the available material (specimen or fragment). Although the composition of cryptofauna varied between closed and open pools (ANOSIM_Open-Closed_; R = 0.40, p = 0.01), only the density of amphipods varied between these habitats (t-test; t = 3.26, p<0.05; Fig 6B), as it was about 30 times higher in open pools. The total density of cryptofauna was positively related to total algal biomass in samples (R^2^ = 0.65; p<0.001; Fig 6C). Algal turfs from closed pools presented higher concentration of soluble sugars and starch content in comparison to open pools, but they did not vary in protein concentration (t-tests; Sugars, t = -2.89, p = 0.014; Starch, t = -7.476, p <0.001; Proteins, t = 0.628, p = 0.538; S3 Fig).

### Feeding pressure and herbivory assays

**Fig 7:**
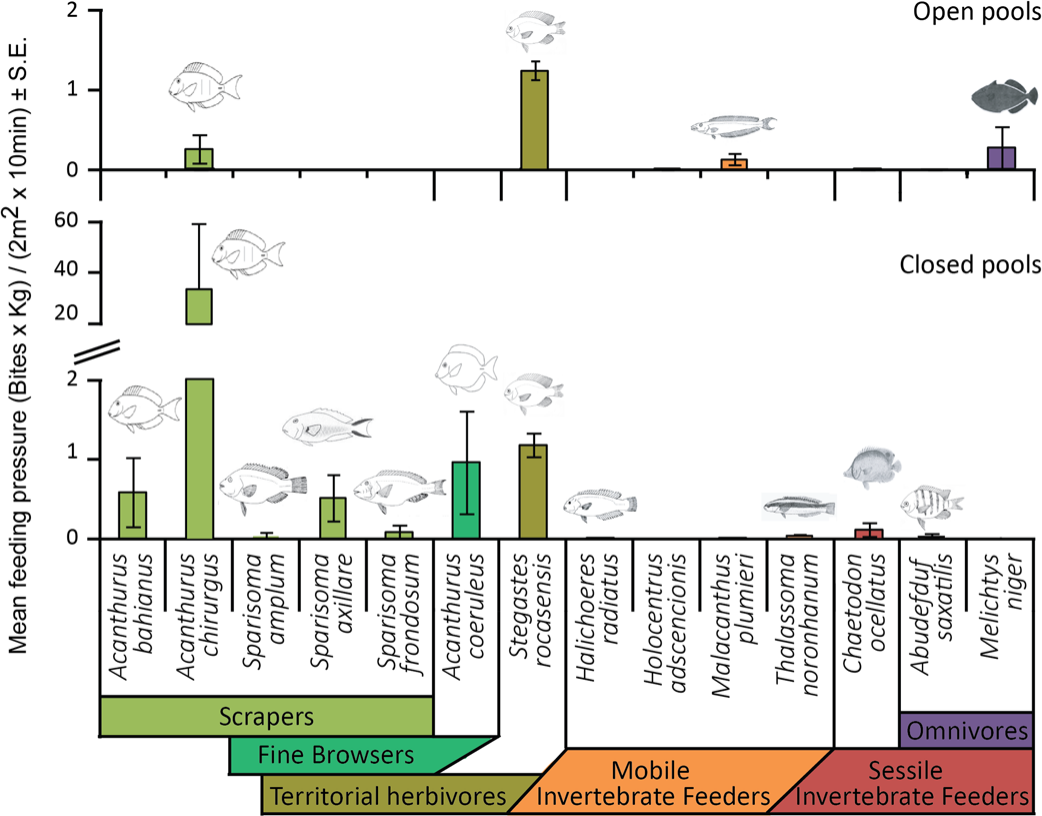
Reef fish feeding pressure on the benthos in closed and open pools. Colored bars indicate species functional groups. Error bars represent standard errors.

Six functional groups, represented by 14 fish species, exerted feeding pressure in closed and open pools, particularly herbivores (scrapers, fine browsers and territorial herbivores; Fig 7). Scrapers performed most of the feeding pressure and represented the highest number of species feeding on the benthos (five). Most of the feeding activity occurred in closed pools, where the total feeding pressure was roughly 20 times higher than in open pools (t-test; t = 2.19, p = 0.03), with the scraper *Acanthurus chirurgus* performing more than 90% (S4 Fig). The fine browser *Acanthurus coeruleus* was recorded feeding on the benthos exclusively in closed pools. The territorial herbivore *Stegastes rocasensis* performed similar feeding pressure in closed and open pools, while mobile and sessile invertebrate feeders, and omnivores exerted a low feeding pressure in both habitats.

**Fig 8:**
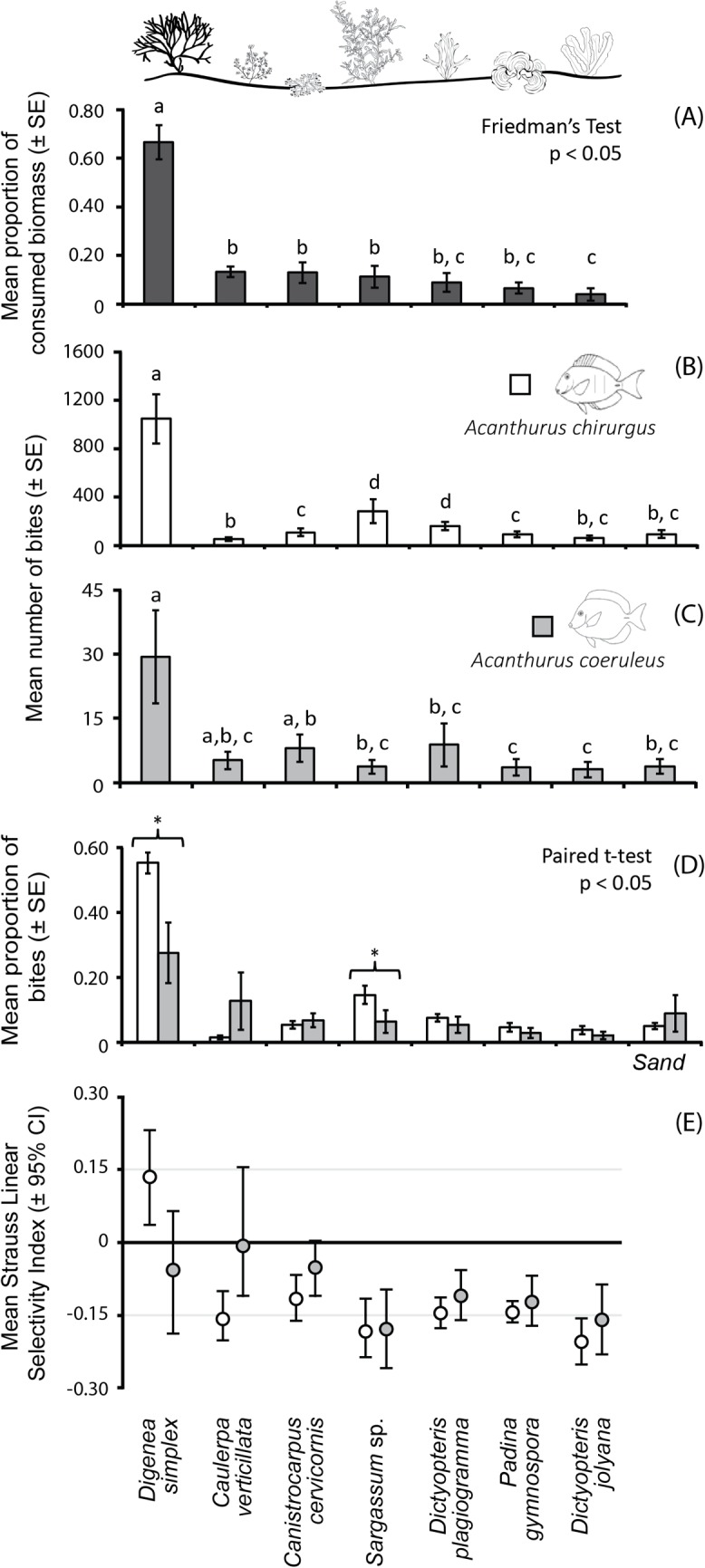
Macroalgal removal and selectivity at the closed pool Tartarugas. (A) Mean proportion of consumed algae biomass; (B) and (C) Mean number of bites on each macroalgae by the herbivorous fishes *Acanthurus chirurgus* and *A*. *coeruleus*, respectively; (D) Mean proportion of bites taken by the two herbivorous species on each algae; (E) Strauss linear selectivity index for the two herbivores on each algae. (*) indicates significant differences at a 5% significance level. In (A), (B), (C) and (D) error bars represent standard errors, and in (E) it represents the 95% confidence interval.

Herbivory assays revealed a higher consumption of the red alga *Digenea simplex* over other six algae (Friedman Test, x^2^ = 37.49, p<0.001; Fig 8A). Only two fish species were recorded removing macroalgae from the experiment: the scraper *A*. *chirurgus* and the fine browser *A*. *coeruleus*. However, most of the algal removal was performed by *A*. *chirurgus*, comprising 95% of the total number of bites recorded in the assays. This species took more bites of *D*. *simplex* than from any other algae, followed by *Sargassum* sp. and *Dictyopteris plagiogramma* (Friedman Test, x^2^ = 45.03, p<0.001; Fig 8B). *A*. *coeruleus* also took a greater number of bites over *D*. *simplex*, but not significantly different from the number of bites over *Caulerpa verticillata* and *Canistrocarpus cervicornis* (Friedman Test, x^2^ = 16.04, p = 0.02; Fig 8C). When comparing the proportion of bites taken by each herbivorous species on each algae, *A*. *chirurgus* contributed to a greater proportion of bites on *D*. *simplex* (Paired t-test, t = 3.19; p<0.05) and *Sargassum* sp. (Paired t-test, t = 2.58; p<0.05) in comparison to *A*. *coeruleus* Fig 8D). The selectivity index indicated that *A*. *chirurgus* significantly selected the macroalgae *D*. *simplex* and avoided the other six algae (Fig 8E). Conversely, *A*. *coeruleus* did not select or avoid *D*. *simplex*, *C*. *verticillata* and *C*. *cervicornis*, but significantly avoided the other four algae (Fig 8D).

**Fig 9:**
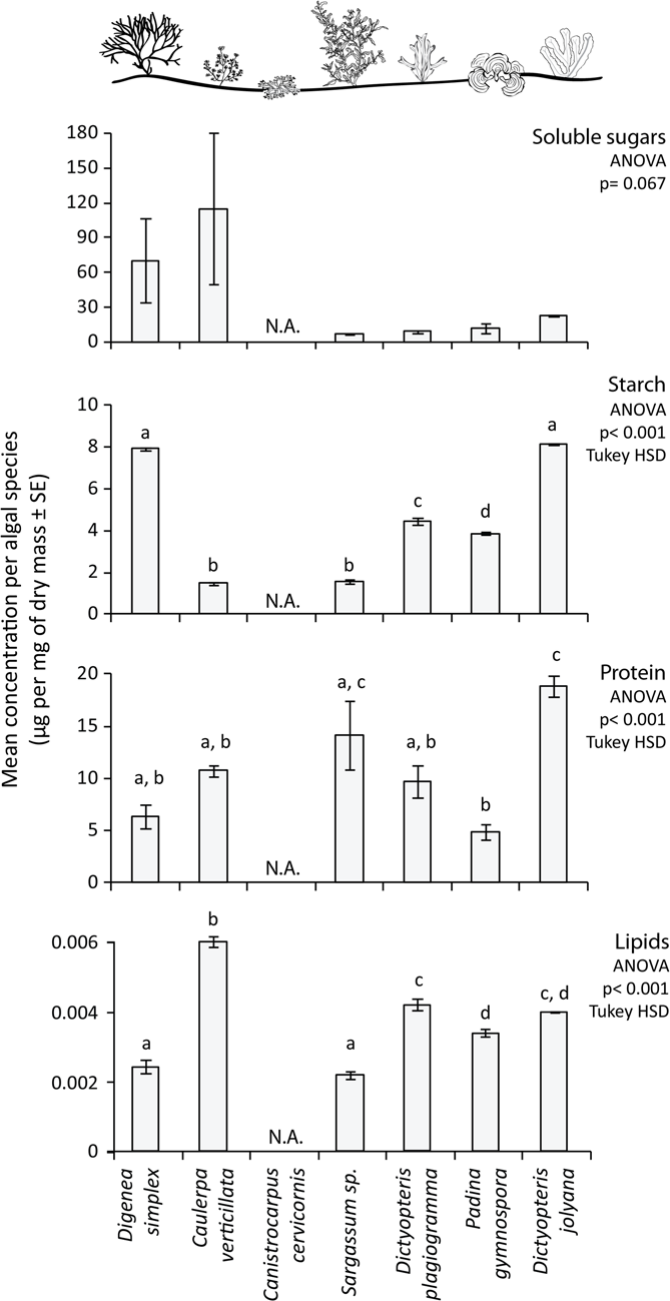
Concentration of soluble sugars, starch, protein and lipid in the algae offered to herbivorous fishes. Letters indicate significant differences according to an analysis of variance followed by a Tukey HSD test. N.A.—not available. Error bars represent standard errors.

The algae used in the assays did not differ in the concentration of soluble sugars but did in starch, protein and lipid concentration (ANOVA, Soluble Sugar: F = 2.327, p = 0.067; Starch: F = 827.900, p< 0.001; Protein: F = 8.641; p< 0.001; Lipids: F = 87.87; p< 0.001; Fig 9). *Digenea simplex* and *Dictyopteris jolyana* presented the highest starch concentration, with the later also presenting the highest protein content, along with *Sargassum* sp. The alga *Caulerpa verticillata* presented the highest lipid concentration, around three times higher than *D*. *simplex*, the most consumed alga in the experiment.

### Diet of herbivorous fishes

**Fig 10:**
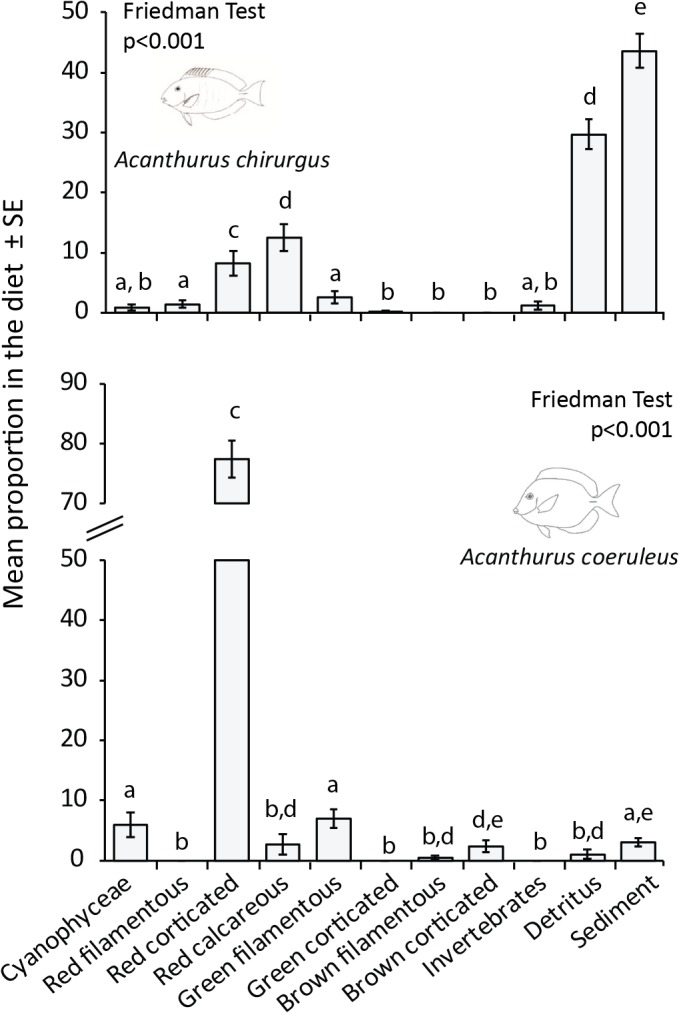
Diet of the two main roving herbivores at Rocas atoll: *Acanthurus chirurgus* and *A*. *coeruleus*. Letters above bars indicate post-hoc comparisons of the Friedman test. Error bars represent standard errors.

The stomach contents of *Acanthurus chirurgus* were dominated by sediment (44%) and detritus (30%), followed by red articulated calcareous algae, mainly *Jania* spp. (12%), and red corticated, especially *Digenea simplex* (8%), with the other components comprising 6% of the diet (Friedman Test, x^2^ = 111.04, p<0.001; Fig 10). On the other hand, the contents of *A*. *coeruleus* were dominated by red corticated algae, especially *D*. *simplex* and *Gelidium* spp. (78%), followed by green filamentous algae (7%) and Cyanophyceae (6%), while detritus (1%) and sediment (3%) presented low abundance (Friedman Test, x^2^ = 74.63, p<0.001; Fig 10). Excluding detritus and sediment, there was a higher proportion of articulated calcareous algae in the contents of *A*. *chirurgus* (48%) followed by red corticated algae (27%), while other items were between 6–12%. Conversely, for *A*. *coeruleus* the dominance of red corticated algae increased to 81% (S8 Table).

## Discussion

As an oceanic system, Rocas atoll is subject to intense wave action and strong tidal currents, which are created as water fills and empties the atoll interior, resulting in an extremely dynamic reef system. In this study, there were remarkable differences in patterns of community structure and feeding pressure on the benthos between closed and open pools. This was evident considering comparative analysis of the reef fish assemblage, benthic cover, the composition, nutritional traits and associated cryptofauna of algal turfs, and fish feeding pressure on the benthos. These differences were probably driven by the distinct hydrodynamic conditions and the related amount of sediment in open and closed pools. Fish feeding pressure, for instance, was more intense in closed pools, which are less exposed in comparison to open pools. Algal turfs were the dominant benthic group in both habitats, however with remarkable differences in composition. Thus, differences in fish feeding pressure could also be related to the higher nutritional quality (e.g. higher sugar and starch contents) of algal turfs in closed pools. These habitats can function as feeding refuges for reef fishes during low tide (e.g. roving herbivores), where they would be more prone to feed because of the protection from intense wave action and currents of the open pools or the outside part of the atoll. Also, closed pools are potentially good refuges against predation, since sharks seem to avoid getting trapped in these environments during low tides and were more common in open pools and outside the atoll. Therefore, fish feeding activities, particularly macroalgal removal, might be determining benthic cover in closed pools by limiting the abundance and restricting the distribution of certain species to less grazed habitats (*e*.*g*., the reef flat and/or open pools). This was particularly evident for *Digenea simplex*, a red corticated macroalgae with low lipid and high soluble sugars concentration. This species was highly consumed in the herbivory assays and figured as an important feeding item in *Acanthurus* spp. stomach contents, but presented low overall abundance or mostly small cropped individuals in both types of pools. Thus, while the benthic patterns observed in open pools seem to be mostly influenced by physical factors, in closed pools the synergy between physical factors and biotic interactions (e.g. reef fish feeding pressure and herbivory) are likely determining its structure and functioning.

### Reef fish assemblages

The reef fish fauna of Brazilian oceanic islands present similar characteristics from the taxonomic and functional perspectives, where the assemblages of Saint Peter and Saint Paul Archipelago are more similar to Trindade Island, while the assemblages of Rocas atoll are remarkably similar to its closest oceanic island, the rocky archipelago of Fernando de Noronha [25]. Rocas atoll and Fernando de Noronha are part of the same volcanic mountain ridge, are roughly 140 km apart, have a common biogeographic history, endemic fish species and similar physical and oceanographic conditions, some of which are different from the other Brazilian oceanic islands [25–28, 69]. From a different perspective, these islands differ in their geomorphology, topography, substrate, sediment composition and habitats [28, 70–72], which could prompt differences in the reef fish assemblages between them. Still, the dominant species in the present study, both in terms of abundance (the planktivore/mobile invertebrate feeder *Thalassoma noronhanum* and the territorial herbivore *Stegastes rocasensis*) and biomass (the scraper *Acanthurus chirurgus*), correspond to the same reported for reef fish assemblages from Fernando de Noronha [73]. Also, overall fish biomass in the present study was comparable to the one reported to Fernando de Noronha [73], albeit largely variable within both localities.

On the other hand, some important aspects of the structure of reef fish assemblages differ between these islands, such as species richness and composition of the macrocarnivore functional group. A recent study on reef fish assemblages of Fernando de Noronha using the same method applied in the present study recorded a total of 80 species [73], while we recorded 66 species at Rocas atoll. It is likely that Fernando de Noronha will present a higher species richness because of a higher diversity of available habitats in comparison to the inner reefs of Rocas atoll. In Fernando de Noronha the small-sized mesopredator *Cephalopholis fulva* was the dominant species [73], while at Rocas atoll larger top predators such as *Lutjanus jocu*, *Ginglymostoma cirratum* and *Negaprion brevirostris* are the main macrocarnivores (Fig 4). Even though all these species are important fishing targets, the dominant species recorded at Rocas atoll are under major threats elsewhere especially due to their large body sizes in comparison to the grouper *C*. *fulva* which is still categorized by the IUCN as “Least Concern” [27, 74–75]. Fishing at Rocas atoll is banned from inside the atoll to depths up to 1,000 m, although some occasional poaching further from the atoll rim still occur due to logistical difficulties to enforce the area. Conversely, most of the coastal waters of Fernando de Noronha are protected by a marine park but only up to the isobaths of 50 m. Additionally, fishing efforts targeting top predators (e.g. sharks) has historically occurred and still occurs close to Fernando de Noronha, where populations of macrocarnivores are potentially more impacted by fishing when compared to populations of Rocas atoll [73, 76].

Reef fish assemblages at Rocas atoll varied between habitats more and less exposed to wave actions (outer reef and open pools, lagoon and closed pools, respectively). Wave exposure interacting with fish swimming abilities can determine the structure and feeding behavior of reef fish assemblages [12, 13–14]. The black triggerfish *Melichthys niger*, for instance, known to inhabit areas with higher wave exposure [73,77], was only recorded in open pools. Conversely, the biomass of the main acanthurid species *A*. *chirurgus* and *A*. *coeruleus* were respectively five and three times higher in closed than in open pools. Similarly, at Laamu Atoll, in the Indian Ocean, assemblages of roving herbivores remarkably varied between habitats inside and outside the atoll rim as a response to wave action [78]. Because at Rocas atoll *A*. *chirurgus* was responsible for 90% of the feeding pressure on the benthos, it is likely that the high hydrodynamic condition of the open pools could limit the feeding behavior of *A*. *chirurgus* and therefore influence the permanency of this species in closed pools. Additionally, reef fish assemblages could also be responding to differences in the benthic cover that could alter for example food and shelter availability (e.g. [13, 73]).

### Benthic cover and algal turfs

In the present study, algal turfs composed between 40 to 55% of benthic cover in tidal pools inside the atoll. The different algal turf composition between open and closed pools determined the differences in overall benthic cover between these habitats. Articulated calcareous algae largely dominated algal turfs of open pools, whereas turfs from closed pools presented a greater contribution of non-calcified algae. The water circulation and the shape of these pools can affect nutrient availability and sediment dynamics [79–80]. Closed pools tend to retain more sediment than open pools, which can cause smothering and shading, reducing the potential primary production [81]. The availability of sediments represents additional abrasion compromising specially those organisms with fleshy composition [82–84]. Conversely, there was a higher abundance of crustose coralline algae in closed pools, which could be related to the relatively higher abundance and feeding pressure of herbivores controlling macroalgae in these habitats [48, 84], or to the tolerance of crustose corallines to burial periods [85]. Similarly, the overall low coral cover at Rocas atoll might be related to the sediment dynamics that, throughout the year, can temporarily burrow coral colonies ([86], Silva *pers*. *obs*.). With increasing disturbance (e.g. exposure conditions, herbivory) algal community structure tends to shift to resistant functional forms, such as turfs and crustose corallines [48, 87].

Algal turfs, forming the epilithical algal matrix, are directly linked to two of the most important trophic pathways for fishes on coral reefs through the consumption of algae, detritus and predation of invertebrates [51, 88–89]. Likewise, at Rocas atoll, algal turfs potentially represent the main trophic pathway between benthic primary production and reef fish consumers irrespective of habitat type. The biomass of algal turfs from open pools was three times higher than in closed pools, with a positive relationship between turf biomass and density of associated cryptofauna. Although this relationship might be simply due to a higher biomass providing more habitat, it might also be favored by the greater structural complexity conferred by articulated coralline algae in comparison to species forming algal turfs in closed pools (*i*.*e*., the same biomass of these types of algae will have different structural complexity). We could also hypothesize that, by selectively feeding on filamentous algae and epiphytes, mesograzers among the cryptofauna promote the dominance of articulated calcareous algae and contribute to the maintenance of a more complex physiognomy in open pools.

The density and composition of cryptofauna associated to algal turfs have been explored between habitats, at different spatial scales, and relative to the volume of particulate matter [90–91]. The ecological and trophic roles of cryptofauna organisms are still poorly understood, but they can be the main protein sources to a variety of mobile invertebrate feeders and even some herbivorous fish [89–93]. Alongside with cryptofauna, organic detritus associated to turf algae further increases the nutritional quality of this substrate [51, 88]. Although detritus load within turfs were not assessed in the present study, it is likely that it followed the pattern identified for sediments (greater amounts in closed pools) because the hydrodynamic of open pools could wash out detritus from the algal matrix. A number of nominally herbivorous fishes are heavily dependent on protein to meet their energetic demands and a large portion of their diets and nutrition is complemented by detritus and invertebrates found within turf algae [93–94]. The identity of seaweeds forming the algal turfs can also play an important role in determining feeding pressure. Some fish might avoid articulated coralline algae because calcified structures can act as physical defenses while other algae can use chemical defenses (see [95–97]). Turf-forming species are specialized for areas subjected to moderate and high grazing pressure and physical stresses (e.g. hydrodynamic), to prevent their competitive exclusion by more productive but less resistant seaweeds [16]. Hence, identifying the algal species that compose algal turfs is critical to understand the trophic pathways involving these assemblages.

### Reef fish feeding pressure on the benthos

Feeding pressure on the benthos was 20 fold greater in closed pools, where algal turfs presented a higher content of soluble sugars and starch than turfs in open pools. Carbohydrates in general are related to energy acquisition in fishes, but the digestion of complex carbohydrates often demands endosymbiontic bacteria that break them down to simpler assimilable components [98]. Most reef fish species present a very limited fermenting capability making soluble sugars and starch the only carbohydrate types possibly used [93]. Both soluble sugar and starch contents can be highly variable, but soluble sugars contents in particular, tend to increase with environmental stress [99]. Thus, the higher soluble sugar content on algal turfs from closed pools could be reinforcing the higher feeding pressure by grazing fishes, creating a stressing environment to the algal turf and a positive feedback between grazing activity and turf sugar content.

Most of the feeding pressure on the benthos was performed by scrapers (especially *Acanthurus chirurgus*), with mobile invertebrate feeders performing a small to negligible feeding pressure. Although this difference could contribute to the lack of relation between feeding pressure and density of cryptofauna, invertebrate feeders usually feed by the end of the day or during the night so their feeding pressure might be underestimated through the applied sampling method [43, 100]. The territorial herbivore *Stegastes rocasensis* performed a similar feeding pressure in both pool types of Rocas atoll and was abundant across the different habitats. Adults of this species were described to use shallow turf-rich areas, while juveniles would inhabit deeper habitats [101]. Damselfishes of the genus *Stegastes* are territorial, small-sized fishes with restricted home ranges [102]. Their intimate association with the substrate allows them to occupy small caves and crevices potentially unaffected or lightly affected by hydrodynamic fluxes.

The dominance of feeding pressure by one species (*A*. *chirurgus*) and functional group (scrapers), could result in low functional redundancy both because there are few species within this group and because feeding pressure is not evenly distributed among them [44]. The important contribution of *A*. *chirurgus* to feeding pressure follows its large abundance and biomass. Closed pools, which contained a particularly high sediment load, concentrated large shoals of this species, regularly seen feeding on sand. Although sediment is known to reduce herbivory pressure on the benthos [103–104], *A*. *chirurgus* is a herbivorous-detritivorous species and sediment is commonly found in its digestive tracts, possibly ingested alongside detritus trapped in algal turfs [65, present work]. The diet of *Acanthurus coeruleus* encompass only 1% detritus [65], and this species was also more abundant in closed than in open pools. This reinforces the hypothesis of closed pools being feeding refuges for reef fishes against intense hydrodynamic conditions (e.g. [14, 16]) or even from predators [105] that are more abundant in open pools and outside the atol rim [29, 76] (Longo, Morais, Silva & Floeter 2014, *pers*. *obs*.). In the present study, lemon sharks (*Negaprion brevirostris*), potential predators of acanthurids, were only recorded in open pools, outside the atoll rim and also swimming towards the pools and the lagoon with the tide inflow [Longo, Morais, Silva & Floeter *pers*. *obs*.]. The presence of apex predators can reduce macroalgal consumption in coral reefs up to ten fold, as a consequence of increased risk-effect to herbivores [105]. Thus, the presence of sharks in open pools and outside the atoll might be leading herbivorous species to feed in closed pools, which contributes to the discrepant feeding pressure between open and closed pools at Rocas atoll. The lower risk-effect caused by less abundant or less threatening predators can result in dramatic changes in fish behavior, potentially affecting the structure of reef fish assemblages and consequently benthic communities [105–106]. In addition, the lower feeding pressure at open pools could also be related the hydrodynamic condition. At more exposed habitats fish usually tend to feed less which can result in lower herbivory pressure [16, 78]. Thus, the observed pattern of feeding pressure is probably a combination between hydrodynamic conditions, presence of predators and nutritional quality of algal turfs which are the main foraging substrate.

### Herbivory assays and diet of the main herbivores

Understanding the patterns of selection or avoidance of algae by fish is a challenging task, since it involves nutritional properties and chemical composition of algae, as well as the ability of fish to properly process the algal material. When seven distinct seaweed species were offered to herbivorous fishes at a closed pool, there was a clear selection of the red algae *Digenea simplex* by the main herbivore *A*. *chirurgus*. Along with *Dictyopteris jolyana*, this species presented the higher content of starches and lower of proteins and lipids among the seaweeds used in the experiment. The avoidance of *Caulerpa verticillata*, *Canistrocarpus cervicornis* and *Dictyopteris* spp. could be related to anti-herbivore chemical defenses in these algae. Caulerpine, caulerpicine and caulerpenyne produced by the genus *Caulerpa* may be feeding deterrent to fish [94, 107] and diterpenoid metabolites produced by *Canistrocarpus* sp. and *Dictyopteris* spp. also present different deterrent effects on fish, urchin and amphipods [108–110]. Although *Sargassum* sp. may have poliphenolics that in sufficient concentration may suppress herbivory by some groups, its structural defense may also play an important role in diminishing susceptibility to herbivory [95, 111–113]. Conversely, *Padina* sp. is known as a palatable macroalgae both in the Pacific [23] and in the Caribbean, where a morphological plasticity in response to high herbivory pressure was documented [114]. This algae was avoided in our herbivory assays possibly because of a lower content of starches and proteins in comparison to *D*. *simplex*, or the presence of highly refractory carbohydrates typical of Phaeophyceae.

A previous study on herbivory at Rocas atoll, demonstrated that *D*. *simplex* was among the most consumed algae, but with no record of the identity of herbivores responsible for algal removal [32]. In our experiment, algal removal was integrally performed by two acanthurid species: *A*. *chirurgus* (95% of the bites) and *A*. *coeruleus*. When accounting for the number of bites, *A*. *chirurgus* was highly dominant generating low functional redundancy [15, 44]. Even considering the remarkable differences in the abundance between these species (S2 Fig), the contribution of *A*. *chirurgus* to algal removal is still disproportionately higher than *A*. *coeruleus*. Regarding the proportion of bites on the algae, these species were fairly redundant, only differing in the proportion of bites taken from *D*. *simplex* and *Sargassum* sp. Although both *Acanthurus* species were important in terms of macroalgal consumption on the assays, a more detailed analysis of their diets revealed the items ingested by these species.

The diet of *A*. *chirurgus* was heavily dominated by sediment and detritus with articulated coralline algae as the most important algal group. On the other hand, *A*. *coeruleus* diet was composed almost 80% with red corticated algae (which includes *D*. *simplex*). At a tropical reef in the Brazilian coast, the diet of *A*. *chirurgus* was dominated by detritus (44%), that only accounted for 1% of *A*. *coeruleus* diet [65]. The differences on food composition between these two species can be related to differences on their food processing modes. Herbivorous-detritivorous species usually possess a thick-walled gizzard-like stomach to mechanically break down ingested material, while browser species usually rely on endosymbiotic fermentation to digest algae [65, 93, 115]. These results indicate some feeding complementarity where *A*. *chirurgus* ingests more articulated calcareous algae, but also redundancy between these species since both ingest red corticated algae. In the Caribbean, *A*. *coeruleus* and *A*. *tractus* were redundant within the genus but complementary to Scarini labrids [116]. At Rocas atoll, the elevated amount of sediment could be benefitting the abundance and feeding pressure of *A*. *chirurgus*. In such a dynamic system, the contribution of more versatile species to ecosystem function can be more important than species diversity itself. Our results indicate that few species dominate important and complex trophic pathways between algal turfs and reef fishes at Rocas atoll, with different levels of complementarity and redundancy.

## Conclusions

This is the first integrative approach describing and quantifying community patterns and ecosystem processes in shallow reef habitats at Rocas atoll and brings up the complexity of this ecosystem. While the patterns and processes observed in the open pools seems to be mostly driven by physical factors and the tolerance of organisms to such conditions, in closed pools there seems to be a synergy between physical factors and biotic interactions (e.g. reef fish feeding pressure and herbivory). The functioning and structure of closed pools are probably a combination of: (1) algal turfs that comprised around 50% of benthic cover and were mostly composed by small cropped pieces of the alga *Digenea simplex*, that has a high nutritional value and was highly consumed in herbivory assays; (2) high abundance of *Acanthurus chirurgus* using these pools as refuges to wave exposure and predation; (3) high feeding pressure of *A*. *chirurgus* affecting the benthic composition of this habitat by cropping *D*. *simplex* and limiting its growth and distribution. Thus, the direct effect of wave exposure interacts with the indirect effect of predators on fish feeding behavior and play an important role in the structure and functioning of these habitats. The relative contribution of direct *vs*. indirect mechanisms shaping biological communities and how they scale-up to ecosystem functioning deserve further attention, particularly on isolated near-pristine systems where natural processes can still be studied under limited human impact.

## Supporting Information

S1 Fig Proportion of abundance and biomass for each reef fish functional group pooling the four studied habitats in Rocas atoll, Brazil. (PDF)

S2 Fig Biomass of the two most abundant herbivorous fishes in the Atoll, *Acanthurus chirurgus* and *A*. *coeruleus*, between closed and open pools. (*) indicate significant differences in the means between habitats (t-test for *A*. *chirurgus*: t = 7.02, p<0.001; t-test for *A*. *coeruleus*: t = 2.16, p<0.05). Error bars represent standard error of the mean.
(PDF)

S3 Fig Mean concentration of soluble sugars, starch and protein in algal turfs of closed and open pools. (*) indicate significant differences (t-tests; Sugars, t = -2.89, p = 0.014; Starch, t = -7.476, p <0.001; Proteins, t = 0.628, p = 0.538). Error bars represent standard error of the mean.
(PDF)

S4 Fig Mean total reef fish feeding pressure on the benthos in closed and open pools. (*) indicate significant differences in the mean feeding pressure between these two habitats (t-test; t = 2.19, p = 0.03). (}) indicate the contribution of the specie *Acanthurus chirurgus* (90% from the total). Error bars represent standard error of the mean.
(PDF)

S1 Table Sample summary of the field effort across the four studied sites in Rocas atoll, Brazil. (DOCX)

S2 Table Analysis of simmilarity (ANOSIM) testing the effect of day on the responses observed for algae biomass loss, and number of bites of *Acanthurus chirurgus* and *A*. *coeruleus* on each algae in the herbivory assays. Tests were based on Bray-Curtis Similarity on square-root transformed data.
(DOCX)

S3 Table Mean density of reef fish species and families recorded across the four studied habitats in the Atoll (open pools, closed pools, lagoon and outer reef). (*) The species *Thalassoma noronhanum* is considered a diurnal planktivore, however regarding the feeding pressure on the benthos this species is acting as a mobile invertebrate feeders, reason why this species is assigned to two functional groups.
(DOCX)

S4 Table Benthic groups recorded in the photoquadrats from open and closed pools of Rocas atoll, Brazil. (DOCX)

S5 Table Summary of t-tests on percent cover of benthic organisms between closed and open pools. Data was square-root transformed prior to the test and significant differences are showed in bold. df = degree of freedom.
(DOCX)

S6 Table Macroalgae groups identified in the algal turfs and their occurrence in the sampled habitats. Groups that polled accounted between 80 and 100% of the samples’ dry weight in closed pools (*) and in open pools (†). Genera and species in bold correspond to the first record of occurrence at Rocas atoll.
(DOCX)

S7 Table Cryptofauna associated to algal turfs and their occurrence in the sampled habitats. (DOCX)

S8 Table Relative abundance of dietary items of the main roving herbivores at Rocas atoll, *Acanthurus chirurgus* and *A*. *coeruleus*. Dominant items are displayed in bold.
(DOCX)
