# Supplementary material for: Between-Habitat Variation of Benthic Cover, Reef Fish Assemblage and Feeding Pressure on the Benthos at the Only Atoll in South Atlantic: Rocas Atoll, NE Brazil
Source: PLoS One. 2015 Jun 10;10(6):e0127176. doi: 10.1371/journal.pone.0127176 (PMC4464550; doi:10.1371/journal.pone.0127176)
Supplement: S1 Fig — (PDF) [file pone.0127176.s001.pdf]

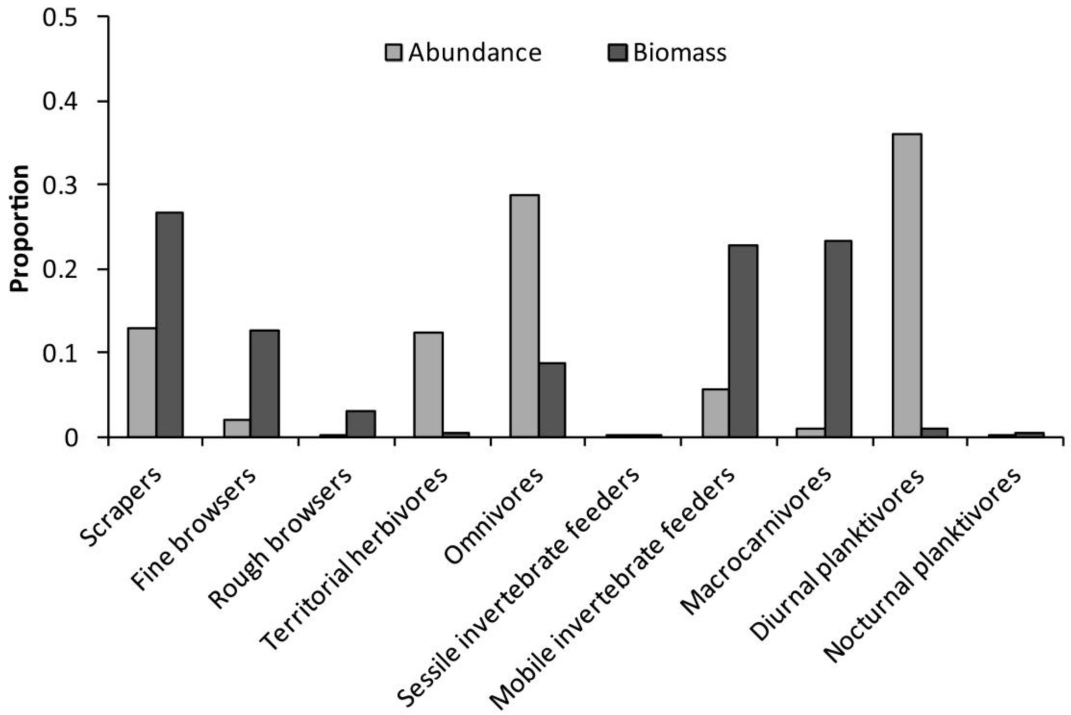

S1 Fig. Proportion of abundance and biomass for each reef fish functional group pooling the four studied habitats in Rocas Atoll, Brazil.
