# Supplementary material for: Between-Habitat Variation of Benthic Cover, Reef Fish Assemblage and Feeding Pressure on the Benthos at the Only Atoll in South Atlantic: Rocas Atoll, NE Brazil
Source: PLoS One. 2015 Jun 10;10(6):e0127176. doi: 10.1371/journal.pone.0127176 (PMC4464550; doi:10.1371/journal.pone.0127176)
Supplement: S2 Fig — (*) indicate significant differences in the means between habitats (t-test for A. chirurgus: t = 7.02, p<0.001; t-test for A. coeruleus: t = 2.16, p<0.05). Error bars represent standard error of the mean. (PDF) [file pone.0127176.s002.pdf]

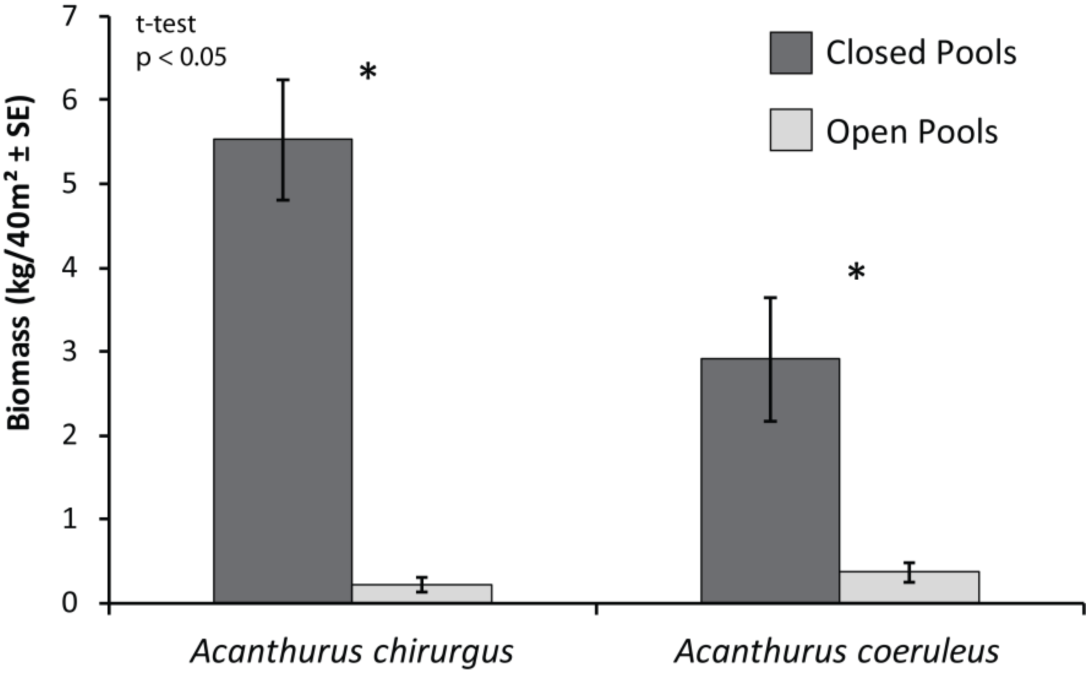

S2 Fig. Biomass of the two most abundant herbivorous fishes in the Atoll, *Acanthurus chirurgus* and *A. coeruleus*, between closed and open pools. (*) indicate significant differences in the means between habitats (t-test for *A. chirurgus*: t=7.02, p<0.001; t-test for *A. coeruleus*: t= 2.16, p<0.05). Error bars represent standard error of the mean.
