# Supplementary material for: Between-Habitat Variation of Benthic Cover, Reef Fish Assemblage and Feeding Pressure on the Benthos at the Only Atoll in South Atlantic: Rocas Atoll, NE Brazil
Source: PLoS One. 2015 Jun 10;10(6):e0127176. doi: 10.1371/journal.pone.0127176 (PMC4464550; doi:10.1371/journal.pone.0127176)
Supplement: S3 Fig — (*) indicate significant differences (t-tests; Sugars, t = -2.89, p = 0.014; Starch, t = -7.476, p <0.001; Proteins, t = 0.628, p = 0.538). Error bars represent standard error of the mean. (PDF) [file pone.0127176.s003.pdf]

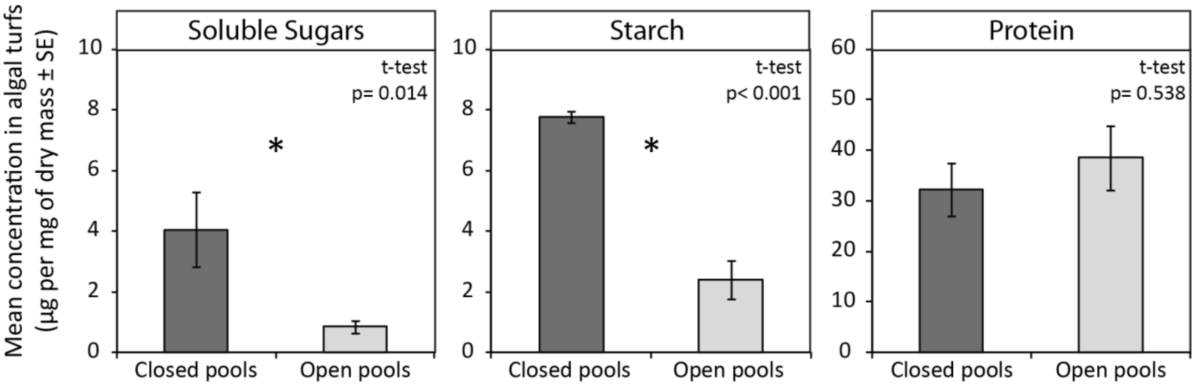

S3 Fig. Mean concentration of soluble sugars, starch and protein in algal turfs of closed and open pools. (*) indicate significant differences (t-tests; Sugars, t = -2.89, p = 0.014; Starch, t = -7.476, p <0.001; Proteins, t = 0.628, p = 0.538). Error bars represent standard error of the mean.
