# Supplementary material for: Between-Habitat Variation of Benthic Cover, Reef Fish Assemblage and Feeding Pressure on the Benthos at the Only Atoll in South Atlantic: Rocas Atoll, NE Brazil
Source: PLoS One. 2015 Jun 10;10(6):e0127176. doi: 10.1371/journal.pone.0127176 (PMC4464550; doi:10.1371/journal.pone.0127176)
Supplement: S4 Fig — (*) indicate significant differences in the mean feeding pressure between these two habitats (t-test; t = 2.19, p = 0.03). (}) indicate the contribution of the specie Acanthurus chirurgus (90% from the total). Error bars represent standard error of the mean. (PDF) [file pone.0127176.s004.pdf]

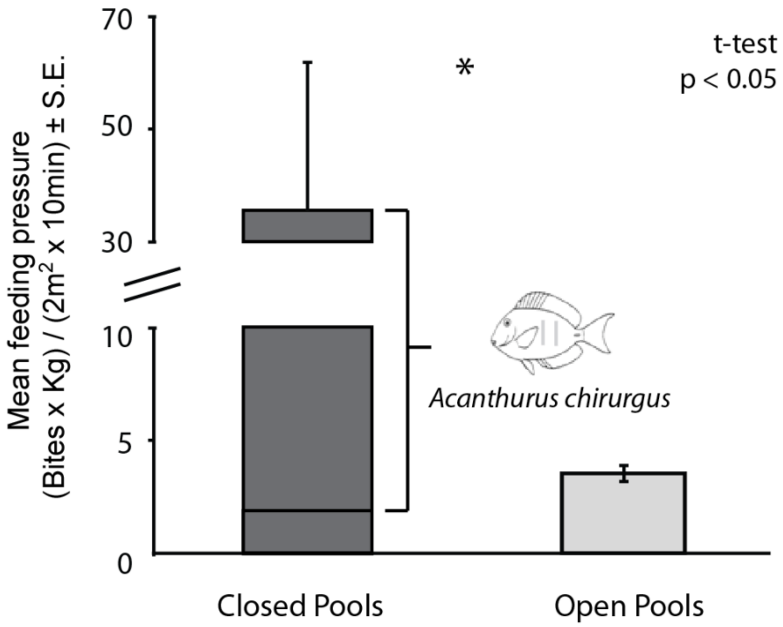

S4 Fig. Mean total reef fish feeding pressure on the benthos in closed and open pools. (*) indicate significant differences in the mean feeding pressure between these two habitats (t-test; t = 2.19, p = 0.03). (}) indicate the contribution of the specie *Acanthurus chirurgus* (90% from the total). Error bars represent standard error of the mean.
