# Supplementary material for: Between-Habitat Variation of Benthic Cover, Reef Fish Assemblage and Feeding Pressure on the Benthos at the Only Atoll in South Atlantic: Rocas Atoll, NE Brazil
Source: PLoS One. 2015 Jun 10;10(6):e0127176. doi: 10.1371/journal.pone.0127176 (PMC4464550; doi:10.1371/journal.pone.0127176)
Supplement: S1 Table — (DOCX) [file pone.0127176.s005.docx]

**S**

**S1 Table.** Sample summary of the field effort across the four studied sites in Rocas Atoll, Brazil.

| **Site** | **Abbreviation** | **Coordinates** | **Depth** | **N**  **Videos** | **N**  **Benthic surveys** | **N**  **Algal turfs** | **N**  **Fish surveys** | **Herbivory assays** |
| --- | --- | --- | --- | --- | --- | --- | --- | --- |
| **Open Pools** |  |  |  |  |  |  |  |  |
| Barretinha | BAR | 03^o^51’75’’ S  33^o^49’05’’ O | 2-3m | - | - | - | 5 | - |
| Falsa Barreta | FBA | 03^o^51’64’’ S  33^o^49’40’’ O | 1-4m | 15 | 18 | 5 | 5 | - |
| Podes Crer | PCR | 03^o^52’20’’ S  33^o^48’45’’ O | 1-3m | 16 | 16 | 5 | 8 | - |
| Salão | SAL | 03^o^52’29’’ S  33^o^48’33’’ O | 8-10m | 9 | 9 | - | 6 | - |
| Sum of samples |  |  |  | **40** | **43** |  | **24** |  |
| **Closed Pools** |  |  |  |  |  |  |  |  |
| Âncoras | ANC | 03^o^52’51’’ S  33^o^48’16’’ O | 2-3m | 15 | 16 | - | 17 | - |
| Cemitério | CEM | 03^o^52’18’’ S  33^o^49’02’’ O | 1-2m | - | - | - | 25 | - |
| Tartarugas | TAR | 03^o^52’43’’ S  33^o^48’59’’ O | 2-3m | 15 | 17 | 5 | 20 | 13 |
| Rocas | ROC | 03^o^51’86’’ S  33^o^47’49’’ O | 1-2m | 15 | 18 | 5 | 20 |  |
| Zulu | ZUL | 03^o^52’33’’ S  33^o^47’89’’ O | 1-2m | - | - | - | 6 | - |
| Sum of samples |  |  |  | **45** | **51** |  | **88** |  |
| **Other habitats** |  |  |  |  |  |  |  |  |
| Lagoon | LAG | 03^o^51’64’’ S  33^o^47’60’’ O | 1-2m | - | - | - | 23 | - |
| Outer reef | OUT | 03^o^29’53’’ S  33^o^49’11’’ O | 10-12m | - | - | - | 21 | - |
| Sum of samples |  |  |  | **-** | **-** | - | **44** | - |
|  |  |  |  |  |  |  |  |  |
| **Total of samples** |  |  |  | **85** | **94** | **20** | **153** | **13** |
