# Supplementary material for: Between-Habitat Variation of Benthic Cover, Reef Fish Assemblage and Feeding Pressure on the Benthos at the Only Atoll in South Atlantic: Rocas Atoll, NE Brazil
Source: PLoS One. 2015 Jun 10;10(6):e0127176. doi: 10.1371/journal.pone.0127176 (PMC4464550; doi:10.1371/journal.pone.0127176)
Supplement: S2 Table — Tests were based on Bray-Curtis Similarity on square-root transformed data. (DOCX) [file pone.0127176.s006.docx]

**S2 Table.** Analysis of similarity (ANOSIM) testing the effect of day on the responses observed for algae biomass loss, and number of bites of *Acanthurus chirurgus* and *A. coeruleus* on each algae in the herbivory assays. Tests were based on Bray-Curtis Similarity on square root transformed data.

| **Differences between days** | **Global Test** | |
| --- | --- | --- |
|  | **R Global** | **p value** |
| **Algae biomass loss** | -0.003 | 0.475 |
| ***Acanthurus chirurgus* (bites)** | 0.024 | 0.321 |
| ***Acanthurus coeruleus* (bites)** | 0.052 | 0.280 |
