# Supplementary material for: Between-Habitat Variation of Benthic Cover, Reef Fish Assemblage and Feeding Pressure on the Benthos at the Only Atoll in South Atlantic: Rocas Atoll, NE Brazil
Source: PLoS One. 2015 Jun 10;10(6):e0127176. doi: 10.1371/journal.pone.0127176 (PMC4464550; doi:10.1371/journal.pone.0127176)
Supplement: S3 Table — (*) The species Thalassoma noronhanum is considered a diurnal planktivore, however regarding the feeding pressure on the benthos this species is acting as a mobile invertebrate feeders, reason why this species is assigned to two functional groups. (DOCX) [file pone.0127176.s007.docx]

**S3 Table.** Mean abundance ± standard error of reef fish species per family recorded across the four studied habitats in the Atoll (closed pools, lagoon, open pools and outer reef). Functional group abbreviations (in order of appearence) stand for: scrp = scrapers, fbrow = fine browsers, minv = mobile invertebrate feeders, npla = nocturnal planktivores, omni = omnivores, mcar = macrocarnivores, ther = territorial herbivores, sinv = sessile invertebrate feeders, dpla = diurnal planktivores, rbrow = rough browsers(*) The species *Thalassoma noronhanum* is considered a diurnal planktivore , however regarding the feeding pressure on the benthos it acts as a mobile invertebrate feeder, reason why it is assigned to two functional groups.

|  |  | **Closed pools** | | | | | **Lagoon** | **Open pools** | | | | **Outer reef** |
| --- | --- | --- | --- | --- | --- | --- | --- | --- | --- | --- | --- | --- |
| **Family/Species** | **Functional group** | **Âncoras** | **Cemitério** | **Rocas pool** | **Tartarugas** | **Zulu** | **Lagoon** | **Barretinha** | **Falsa barreta** | **Podes crer** | **Salão** | **Outer reef** |
|  |  |  |  |  |  |  |  |  |  |  |  |  |
| **Acanthuridae** |  |  |  |  |  |  |  |  |  |  |  |  |
| *Acanthurus bahianus* | scrp | 0.06 ± 0.06 | - | - | - | - | - | - | - | - | - | - |
| *Acanthurus chirurgus* | scrp | 68.41 ± 15.69 | 20.68 ± 6.26 | 68.2 ± 7.46 | 33 ± 7.19 | 82.67 ± 22.85 | 26.3 ± 5.09 | 24.4 ± 14.37 | 2.2 ± 1.11 | 4.5 ± 1.79 | 0.5 ± 0.34 | 19.67 ± 3.22 |
| *Acanthurus coeruleus* | fbrow | 10.47 ± 4 | 1.2 ± 0.51 | 4.65 ± 2.88 | 16.05 ± 4.71 | 4 ± 2.82 | 1.39 ± 0.85 | 2.6 ± 1.25 | 3.4 ± 0.93 | 0.63 ± 0.42 | 0.83 ± 0.48 | 9.9 ± 2.99 |
| **Albulidae** |  |  |  |  |  |  |  |  |  |  |  |  |
| *Albula vulpes* | minv | - | - | - | - | - | - | 144 ± 66.98 | - | - | - | - |
| **Apogonidae** |  |  |  |  |  |  |  |  |  |  |  |  |
| *Apogon americanus* | npla | - | 0.12 ± 0.09 | 0.05 ± 0.05 | - | - | - | - | - | - | 0.17 ± 0.17 | - |
| **Balistidae** |  |  |  |  |  |  |  |  |  |  |  |  |
| *Balistes vetula* | minv | - | - | - | - | - | 0.17 ± 0.1 | - | - | - | - | - |
| *Melichthys niger* | omni | - | - | - | 0.05 ± 0.05 | - | - | - | - | 8.63 ± 2.23 | 9.83 ± 4.77 | - |
| **Belonidae** |  |  |  |  |  |  |  |  |  |  |  |  |
| *Strongylura timucu* | mcar | 0.29 ± 0.29 | 0.16 ± 0.16 | - | - | - | 0.43 ± 0.31 | - | - | 0.25 ± 0.25 | - | - |
| **Blenniidae** |  |  |  |  |  |  |  |  |  |  |  |  |
| *Ophioblennius trinitatis* | ther | 0.06 ± 0.06 | - | 0.15 ± 0.11 | - | 0.17 ± 0.17 | 0.39 ± 0.26 | - | - | - | - | - |
| **Bothidae** |  |  |  |  |  |  |  |  |  |  |  |  |
| *Bothus lunatus* | minv | 0.06 ± 0.06 | - | - | - | - | - | - | - | - | - | - |
| **Carangidae** |  |  |  |  |  |  |  |  |  |  |  |  |
| *Carangoides bartholomaei* | mcar | 1.06 ± 0.42 | 1.08 ± 0.73 | 0.6 ± 0.46 | 0.65 ± 0.31 | - | 0.61 ± 0.4 | 0.8 ± 0.49 | - | 0.25 ± 0.16 | - | 0.1 ± 0.07 |
| *Caranx crysos* | mcar | - | - | - | - | - | - | - | - | - | - | 0.1 ± 0.1 |
| *Caranx latus* | mcar | - | - | - | 0.1 ± 0.07 | - | 0.04 ± 0.04 | - | - | - | - | - |
| *Caranx lugubris* | mcar | - | - | - | - | - | - | - | - | - | - | 0.19 ± 0.11 |
| **Carcharhinidae** |  |  |  |  |  |  |  |  |  |  |  |  |
| *Carcharhinus perezii* | mcar | - | - | - | - | - | - | - | - | - | - | 0.05 ± 0.05 |
| *Negaprion brevirostris* | mcar | - | - | 0.05 ± 0.05 | - | - | - | 0.2 ± 0.2 | - | 0.13 ± 0.13 | - | - |
| **Chaetodontidae** |  |  |  |  |  |  |  |  |  |  |  |  |
| *Chaetodon ocellatus* | sinv | 1.71 ± 0.5 | 0.12 ± 0.07 | 1.35 ± 0.28 | 0.1 ± 0.1 | 1.83 ± 0.48 | 0.43 ± 0.14 | - | - | - | - | 0.1 ± 0.1 |
| **Diodontidae** |  |  |  |  |  |  |  |  |  |  |  |  |
| *Diodon hystrix* | minv | - | - | 0.05 ± 0.05 | - | - | - | - | - | - | - | - |
| **Epinephelidae** |  |  |  |  |  |  |  |  |  |  |  |  |
| *Cephalopholis fulva* | mcar | 1.88 ± 0.84 | 0.04 ± 0.04 | 2.9 ± 0.79 | - | 2.33 ± 1.17 | 4.74 ± 0.68 | - | - | - | 0.17 ± 0.17 | 0.05 ± 0.05 |
| *Dermatolepis inermis* | mcar | - | - | - | - | - | - | - | - | - | 0.17 ± 0.17 | 0.1 ± 0.07 |
| *Paranthias furcifer* | dpla | - | - | - | - | - | 0.39 ± 0.27 | - | - | - | - | - |
| **Ginglymostomatidae** |  |  |  |  |  |  |  |  |  |  |  |  |
| *Ginglymostoma cirratum* | mcar | - | 0.16 ± 0.11 | 0.15 ± 0.15 | - | 0.17 ± 0.17 | 0.04 ± 0.04 | - | - | - | - | - |
| **Gobiidae** |  |  |  |  |  |  |  |  |  |  |  |  |
| *Coryphopterus* sp. | omni | 40.88 ± 11.44 | 87.88 ± 8.73 | 40.5 ± 4.14 | 57.15 ± 11.1 | 20.67 ± 9.04 | 55.26 ± 9.18 | - | 6 ± 1.52 | 0.75 ± 0.75 | 1.83 ± 0.83 | 0.05 ± 0.05 |
| *Elacatinus phthirophagus* | minv | - | - | - | 0.05 ± 0.05 | - | - | - | - | - | - | - |
| *Gnatholepis thompsoni* | omni | 6.35 ± 1.87 | 71.52 ± 10.01 | 8.8 ± 1.73 | 30.8 ± 7.33 | 1.67 ± 1.67 | 10.43 ± 2.6 | - | 3.6 ± 0.81 | 7.25 ± 3.51 | - | 0.05 ± 0.05 |
| **Haemulidae** |  |  |  |  |  |  |  |  |  |  |  |  |
| *Haemulon chrysargyreum* | minv | 12.94 ± 12.94 | - | - | - | 56.17 ± 37.4 | - | - | - | - | - | 0.52 ± 0.48 |
| *Haemulon parra* | minv | - | - | 3.7 ± 1.4 | - | 20.17 ± 10.89 | 0.3 ± 0.26 | - | - | - | - | 0.1 ± 0.1 |
| **Holocentridae** |  |  |  |  |  |  |  |  |  |  |  |  |
| *Holocentrus adscensionis* | minv | 1.94 ± 1.27 | 9.16 ± 3.56 | 9.25 ± 3.58 | 0.85 ± 0.25 | 0.17 ± 0.17 | 3.87 ± 1.74 | 2.4 ± 1.03 | 9.8 ± 2.22 | 7 ± 2.57 | 2.33 ± 1.56 | 0.52 ± 0.15 |
| *Myripristis jacobus* | npla | - | 0.52 ± 0.44 | - | - | 0.17 ± 0.17 | 0.43 ± 0.31 | - | - | - | - | 0.48 ± 0.2 |
| **Kyphosidae** |  |  |  |  |  |  |  |  |  |  |  |  |
| *Kyphosus* spp. | rbrow | - | - | 1.25 ± 1.1 | - | - | - | - | 0.4 ± 0.4 | 0.5 ± 0.5 | - | 1.38 ± 0.71 |
| **Labridae** |  |  |  |  |  |  |  |  |  |  |  |  |
| *Halichoeres radiatus* | minv | 1.24 ± 0.3 | 1.52 ± 0.99 | 2.6 ± 0.46 | 0.15 ± 0.08 | 0.83 ± 0.4 | 1.39 ± 0.45 | 4 ± 2.59 | 2.4 ± 0.68 | - | 1.83 ± 0.65 | 7.43 ± 1.1 |
| *Sparisoma amplum* | scrp | 1 ± 0.39 | - | - | 0.05 ± 0.05 | 1.17 ± 0.6 | - | - | - | - | - | - |
| *Sparisoma axillare* | scrp | 1.29 ± 0.43 | 0.28 ± 0.24 | 17.7 ± 3.54 | - | 1.17 ± 0.83 | 0.87 ± 0.35 | - | - | - | - | - |
| *Sparisoma frondosum* | scrp | 0.06 ± 0.06 | - | 0.05 ± 0.05 | 0.05 ± 0.05 | 0.83 ± 0.83 | - | - | - | - | - | 0.1 ± 0.07 |
| *Sparisoma radians* | scrp | - | - | - | - | - | - | - | - | - | - | 0.24 ± 0.12 |
| *Sparisoma* sp. | scrp | 0.06 ± 0.06 | - | - | - | - | - | - | - | - | - | 0.05 ± 0.05 |
| *Thalassoma noronhanum** | dpla/minv | 141.12 ± 30.86 | 75.96 ± 9.51 | 14.2 ± 6.94 | 134.45 ± 20.91 | 57 ± 28.4 | 46.35 ± 7.88 | 157.6 ± 29.91 | 105.8 ± 13.04 | 202.25 ± 24.07 | 80.5 ± 20.3 | 171.86 ± 20.62 |
| **Lutjanidae** |  |  |  |  |  |  |  |  |  |  |  |  |
| *Lutjanus jocu* | mcar | 0.18 ± 0.13 | 1.08 ± 0.62 | 1.6 ± 1.13 | 0.6 ± 0.22 | 0.17 ± 0.17 | 0.13 ± 0.07 | - | 0.2 ± 0.2 | 0.13 ± 0.13 | 0.17 ± 0.17 | 0.1 ± 0.07 |
| **Malacanthidae** |  |  |  |  |  |  |  |  |  |  |  |  |
| *Malacanthus plumieri* | mcar | 0.29 ± 0.11 | 0.48 ± 0.13 | 0.1 ± 0.07 | 0.95 ± 0.2 | - | 0.26 ± 0.11 | 0.8 ± 0.37 | - | 0.25 ± 0.16 | - | - |
|  |  |  |  |  |  |  |  |  |  |  |  |  |
| **Monacanthidae** |  |  |  |  |  |  |  |  |  |  |  |  |
| *Aluterus scriptus* | sinv | - | - | - | 0.05 ± 0.05 | - | - | - | - | - | - | - |
| **Mullidae** |  |  |  |  |  |  |  |  |  |  |  |  |
| *Mulloidichthys martinicus* | minv | 0.12 ± 0.12 | 0.68 ± 0.48 | - | 1 ± 0.69 | - | 0.43 ± 0.43 | - | - | - | 0.17 ± 0.17 | 0.52 ± 0.22 |
| *Pseudupeneus maculatus* | minv | - | - | 0.1 ± 0.1 | - | - | 0.04 ± 0.04 | - | - | - | 0.17 ± 0.17 | 0.05 ± 0.05 |
| **Muraenidae** |  |  |  |  |  |  |  |  |  |  |  |  |
| *Gymnothorax miliaris* | mcar | - | - | 0.05 ± 0.05 | 0.05 ± 0.05 | - | - | - | - | - | - | - |
| *Muraena pavonina* | mcar | - | - | - | - | 0.17 ± 0.17 | - | - | - | - | - | - |
| **Ostraciidae** |  |  |  |  |  |  |  |  |  |  |  |  |
| *Acanthostracion polygonius* | sinv | - | - | - | - | - | - | - | - | - | - | 0.05 ± 0.05 |
| *Lactophrys trigonus* | minv | - | - | 0.05 ± 0.05 | - | - | - | - | - | - | - | - |
| **Pempheridae** |  |  |  |  |  |  |  |  |  |  |  |  |
| *Pempheris schomburgkii* | npla | - | - | - | - | - | - | - | - | - | - | 3.81 ± 1.86 |
| **Pomacanthidae** |  |  |  |  |  |  |  |  |  |  |  |  |
| *Pomacanthus paru* | omni | 0.06 ± 0.06 | 0.04 ± 0.04 | 0.05 ± 0.05 | 0.05 ± 0.05 | - | 0.04 ± 0.04 | - | - | - | 0.83 ± 0.48 | 0.33 ± 0.11 |
| **Pomacentridae** |  |  |  |  |  |  |  |  |  |  |  |  |
| *Abudefduf saxatilis* | omni | 27.88 ± 13.27 | 17.36 ± 4.56 | 21.95 ± 6.38 | 69.15 ± 21.67 | 7.83 ± 1.78 | 9.52 ± 3.61 | 28.8 ± 9.32 | 3 ± 2.28 | 7.13 ± 3.5 | 4.5 ± 3.51 | 14.62 ± 2.79 |
| *Chromis multilineata* | dpla | 14.94 ± 8.88 | - | 8.1 ± 4.36 | 0.2 ± 0.16 | 1.83 ± 1.33 | 3.7 ± 1.87 | - | - | - | 0.33 ± 0.33 | 0.1 ± 0.1 |
| *Stegastes pictus* | ther | - | - | - | - | - | 0.26 ± 0.11 | - | - | - | - | - |
| *Stegastes rocasensis* | ther | 27.88 ± 4.46 | 39.76 ± 4.27 | 15.4 ± 3.16 | 50.3 ± 6.2 | 20.83 ± 5.56 | 28.3 ± 3.71 | 59.6 ± 14.25 | 87 ± 1.1 | 105.75 ± 14.62 | 61.17 ± 14.87 | 2 ± 0.38 |
| **Serranidae** |  |  |  |  |  |  |  |  |  |  |  |  |
| *Rypticus saponaceus* | mcar | - | 0.04 ± 0.04 | - | - | 0.17 ± 0.17 | - | - | - | - | - | 0.05 ± 0.05 |
|  |  |  |  |  |  |  |  |  |  |  |  |  |
| **Sphyraenidae** |  |  |  |  |  |  |  |  |  |  |  |  |
| *Sphyraena barracuda* | mcar | 0.06 ± 0.06 | - | - | 0.05 ± 0.05 | - | - | - | - | - | - | - |
