# Supplementary material for: Between-Habitat Variation of Benthic Cover, Reef Fish Assemblage and Feeding Pressure on the Benthos at the Only Atoll in South Atlantic: Rocas Atoll, NE Brazil
Source: PLoS One. 2015 Jun 10;10(6):e0127176. doi: 10.1371/journal.pone.0127176 (PMC4464550; doi:10.1371/journal.pone.0127176)
Supplement: S4 Table — (DOCX) [file pone.0127176.s008.docx]

**S4 Table.** Benthic groups recorded in the photoquadrats from open and closed pools of Rocas Atoll, Brazil.

| **Benthic groups** | | | **Closed Pools** | | | **Open Pools** | | |
| --- | --- | --- | --- | --- | --- | --- | --- | --- |
|  |  |  | **Âncoras** | **Rocas** | **Tartarugas** | **Falsa**  **Barreta** | **Podes**  **Crer** | **Salão** |
| **Phylum Porifera** | | |  |  |  |  |  |  |
|  | Encrusting form | | X | X | X | X | X |  |
|  | Massive form | | X | X |  |  |  |  |
|  | Tubular form | |  | X |  |  |  |  |
| **Phylum Cnidaria** | | |  |  |  |  |  |  |
|  | Class Anthozoa | |  |  |  |  |  |  |
|  | Order Zoanthidea | |  |  |  |  |  |  |
|  | | *Palythoa caribaeorum* | X |  |  |  |  | X |
|  | | *Zoanthus sociatus* | X |  |  | X | X |  |
| Order Scleractinia | | |  |  |  |  |  |  |
|  | | *Favia gravida* |  | X |  |  |  |  |
|  | | *Mussismilia hispida* | X |  |  |  |  |  |
|  | | *Porites astreoides* | X | X | X |  |  |  |
|  | | *Siderastrea stellata* | X | X | X | X | X | X |
| **Phylum Chordata** | | |  |  |  |  |  |  |
|  | Class Ascidiacea | | X |  |  |  |  |  |
| **Algal turfs** | | |  |  |  |  |  |  |
|  | | Calcareous turf | X | X |  | X | X | X |
|  | | Non-calcified turf | X | X | X | X | X | X |
| **Articulated Calcareous Algae** | | |  | X |  | X |  |  |
|  | | *Tricleocarpa cilyndrica* |  |  |  |  |  | X |
|  | | *Galaxaura* sp. | X |  |  | X |  | X |
| **Cyanobacteria (microfilm)** | | | X | X | X | X | X |  |
| **Corticated Macroalgae** | | |  |  |  |  |  |  |
|  | | *Champia parvula* |  |  | X |  |  |  |
|  | | *Codium* spp. |  | X |  |  |  | X |
|  | | *Digenia simplex* |  | X |  |  |  |  |
|  | | *Hypnea musciformis* |  |  |  | X | X |  |
|  | | Non-identified | X | X |  | X | X |  |
| **Crustose algae** | | |  |  |  |  |  |  |
|  | | Crustose coralline algae | X | X |  | X | X |  |
|  | | Other  non-calcified algae | X |  | X |  |  |  |
| **Filamentous algae** | | |  |  |  |  |  |  |
|  | | *Bryopsis pennata* |  |  |  |  | X | X |
|  | | *Caulerpa* sp. |  |  |  | X |  |  |
|  | | *Caulerpa verticillata* |  |  |  | X |  | X |
|  | | *Chaetomorpha* sp. |  |  |  | X |  |  |
|  | | Non-identified | X | X |  | X | X |  |
| **Foliose macroalgae** | | |  |  |  |  |  |  |
|  | | *Canistrocarpus* sp. |  |  |  | X | X | X |
|  | | *Dictyopteris* sp. |  |  |  | X | X |  |
| **Leathery macroalgae** | | |  |  |  |  |  |  |
|  | | *Lobophora variegata* | X |  |  |  |  |  |
|  | | *Padina* sp |  |  |  |  | X |  |
|  | | *Sargassum* spp. |  | X |  | X | X |  |
|  | | Non-identified |  |  |  |  | X |  |
| **Sand and sediment** | | | X | X | X | X | X | X |
