# Supplementary material for: Between-Habitat Variation of Benthic Cover, Reef Fish Assemblage and Feeding Pressure on the Benthos at the Only Atoll in South Atlantic: Rocas Atoll, NE Brazil
Source: PLoS One. 2015 Jun 10;10(6):e0127176. doi: 10.1371/journal.pone.0127176 (PMC4464550; doi:10.1371/journal.pone.0127176)
Supplement: S5 Table — Data was square-root transformed prior to the test and significant differences are showed in bold. df = degree of freedom. (DOCX) [file pone.0127176.s009.docx]

**S5 Table.** Summary of t-tests on percent cover of benthic organisms between closed and open pools. Data was square root transformed prior to the test and significant differences are showed in bold. **df** = degree of freedom.

| **Benthic Group** | **t-value** | **df** | **p value** | **Between pools** |
| --- | --- | --- | --- | --- |
| Non-calcified turf | 6.29 | 97 | **< 0.001** | Closed > Open |
| Calcareous turf | -10.17 | 97 | **< 0.001** | Closed < Open |
| Sediment | 4.51 | 97 | **< 0.001** | Closed > Open |
| *Caulerpa verticilatta* | -6.00 | 97 | **< 0.001** | Closed < Open |
| *Siderastrea* spp. | -3.22 | 97 | **0.001** | Closed < Open |
| *Zoanthus* sp. | -3.74 | 97 | **< 0.001** | Closed < Open |
| Microfilm (cyanobacteria) | 2.03 | 97 | **< 0.05** | Closed > Open |
| *Sargassum* sp. | -3.80 | 97 | **< 0.001** | Closed < Open |
| *Canistrocarpus* sp. | -4.64 | 97 | **< 0.001** | Closed < Open |
| Calcareous Coralline Algae | 2.42 | 97 | **p< 0.05** | Closed > Open |
