# Supplementary material for: Between-Habitat Variation of Benthic Cover, Reef Fish Assemblage and Feeding Pressure on the Benthos at the Only Atoll in South Atlantic: Rocas Atoll, NE Brazil
Source: PLoS One. 2015 Jun 10;10(6):e0127176. doi: 10.1371/journal.pone.0127176 (PMC4464550; doi:10.1371/journal.pone.0127176)
Supplement: S6 Table — Groups that polled accounted between 80 and 100% of the samples’ dry weight in closed pools (*) and in open pools (†). Genera and species in bold correspond to the first record of occurrence at Rocas atoll. (DOCX) [file pone.0127176.s010.docx]

**S6 Table.** Macroalgae groups identified in the algal turfs and their occurrence in the sampled habitats. Groups that polled accounted between 80 and 100% of the samples’ dry weight in closed pools (*) and in open pools (†). Genera and species in bold correspond to the first record of occurrence at Rocas Atoll.

| **Macroalgae groups** | **Closed Pools** | | **Open Pools** | |
| --- | --- | --- | --- | --- |
|  | **Rocas** | **Tartarugas** | **Falsa Barreta** | **Podes Crer** |
| **Division Rhodophyta** |  |  |  |  |
| *Acrochaetium* sp. | X |  |  |  |
| *Amphiroa* sp. **†** |  |  | X | X |
| *Bryothamnion triquetrum* |  |  | X |  |
| *Ceramium gracilimum* |  |  |  | X |
| ***Chondria polyrhiza* *** | **X** | **X** | **X** | **X** |
| ***Chondria* sp.** |  |  |  | **X** |
| *Digenea simplex* * | X | X | X |  |
| *Erythrocladia* sp. |  |  |  |  |
| *Erythrotrichia* sp. |  |  |  |  |
| *Gelidiella acerosa* |  |  | X |  |
| *Gelidiella* sp. | X |  |  |  |
| *Gelidium americanum* |  |  | X |  |
| ***Gelidium crinale* *** | **X** | **X** | **X** | **X** |
| *Gelidium pusillum* | X | X |  |  |
| *Gelidium* sp. | X |  |  |  |
| ***Haliptilon subulatum*** |  |  | **X** |  |
| ***Hypnea cenomyce*** |  |  | **X** | **X** |
| *Hypnea musciformis* | X |  | X |  |
| *Jania adhaerens* | X | X |  | X |
| *Jania capillacea* |  |  |  | X |
| ***Jania verrucosa*** |  |  | **X** |  |
| ***Jania prolifera*** |  | **X** |  |  |
| *Jania* sp. **†** |  |  | X | X |
| *Polysiphonia* sp. |  | X |  |  |
| *Polysiphonia subtilissima* |  | X |  |  |
| ***Pterocladiella sanctarum*** |  | **X** | **X** | **X** |
| **Division Chlorophyta** |  |  |  |  |
| *Bryopsis* sp. |  | X |  |  |
| *Caulerpa mexicana* |  |  | X |  |
| *Caulerpa verticillata* |  |  | X |  |
| ***Caulerpella ambigua*** |  |  | **X** | **X** |
| ***Chaetomorpha spiralis*** |  |  | **X** |  |
| *Cladophora* sp. | X | X |  | X |
| *Derbesia marina* | X |  |  | X |
| *Valonia aegagropila* **†** | X | X | X |  |
| **Division Heterokontophyta** |  |  |  |  |
| **Class Phaeophyceae** |  |  |  |  |
| *Canistrocarpus cervicornis* |  |  |  | X |
| *Dictyopteris delicatula* |  | X | X | X |
| ***Dictyopteris plagiogramma*** | **X** | **X** |  |  |
| *Dictyopteris* sp. 1 |  |  | X | X |
| *Dictyopteris* sp. 2 | X |  |  |  |
| *Dictyota mertensii* |  |  |  | X |
| *Dictyota pulchella* |  |  | X | X |
| *Ectocarpus* sp. |  |  | X |  |
| ***Levringia* sp. *** | **X** |  |  |  |
| *Lobophora* sp. |  |  | X |  |
| *Padina* sp. |  |  | X |  |
| *Sargassum* sp. |  |  | X |  |
| *Sphacelaria* sp. |  | X | X | X |
