# Supplementary material for: Between-Habitat Variation of Benthic Cover, Reef Fish Assemblage and Feeding Pressure on the Benthos at the Only Atoll in South Atlantic: Rocas Atoll, NE Brazil
Source: PLoS One. 2015 Jun 10;10(6):e0127176. doi: 10.1371/journal.pone.0127176 (PMC4464550; doi:10.1371/journal.pone.0127176)
Supplement: S7 Table — (DOCX) [file pone.0127176.s011.docx]

**S7 Table.** Cryptofauna associated to algal turfs and their occurrence in the sampled habitats.

| **Groups** | **Closed Pools** | | **Open Pools** | |
| --- | --- | --- | --- | --- |
|  | **Rocas** | **Tartarugas** | **Falsa Barreta** | **Podes Crer** |
| **Phylum Annelida** |  |  |  |  |
| Class Polychaeta | X | X |  | X |
| **Phylum Arthropoda** |  |  |  |  |
| **Subphylum Crustacea** |  |  |  |  |
| Class Malacostraca |  |  |  |  |
| Order Amphipoda |  |  |  |  |
| Morpho 1 |  | X | X | X |
| Morpho 2 |  |  | X | X |
| Morpho 3 |  |  | X | X |
| Morpho 4 |  | X |  | X |
| Morpho 5 | X |  | X | X |
| Order Decapoda |  |  |  |  |
| Family Mithracidae |  |  |  |  |
| Morpho 1 | X |  | X | X |
| Morpho 2 | X |  |  | X |
| Morpho 3 |  |  |  | X |
| Super Family Xanthoidea |  |  |  |  |
| Morpho 1 | X |  |  |  |
| Order Isopoda |  |  |  |  |
| Morpho 1 |  |  |  | X |
| Order Tanaidacea |  |  |  |  |
| Morpho 1 |  | X | X | X |
| Morpho 2 |  | X | X | X |
| **Subphylum Hexapoda** |  |  |  |  |
| Class Insecta |  |  |  |  |
| Order Diptera |  |  |  |  |
| Family Chironomidae |  |  |  |  |
| Larvae | X | X | X |  |
| **Phylum Cnidaria** |  |  |  |  |
| Class Anthozoa |  |  |  |  |
| Order Zoantharia |  |  |  |  |
| Family Zoanthidae |  |  |  |  |
| *Zoanthus sociatus* |  | X | X | X |
| **Phylum Echinodermata** |  |  |  |  |
| Class Ophiuroidea |  |  |  |  |
| Morpho 1 |  |  |  | X |
| Morpho 2 |  |  |  | X |
| Morpho 3 |  |  |  | X |
| **Phyllum Mollusca** |  |  |  |  |
| Class Gastropoda |  |  |  |  |
| Morpho 1 |  | X |  |  |
| Morpho 2 |  | X |  |  |
| Morpho 3 | X |  |  |  |
