# Supplementary material for: Between-Habitat Variation of Benthic Cover, Reef Fish Assemblage and Feeding Pressure on the Benthos at the Only Atoll in South Atlantic: Rocas Atoll, NE Brazil
Source: PLoS One. 2015 Jun 10;10(6):e0127176. doi: 10.1371/journal.pone.0127176 (PMC4464550; doi:10.1371/journal.pone.0127176)
Supplement: S8 Table — Dominant items are displayed in bold. (DOCX) [file pone.0127176.s012.docx]

**S8 Table.** Relative abundance of dietary items, excluding sediment and detritus, of the main roving herbivores at Rocas Atoll, *Acanthurus chirurgus* and *A. coeruleus*. Dominant items are displayed in bold.

|  | ***Acanthurus chirurgus*** | ***Acanthurus coeruleus*** | |
| --- | --- | --- | --- |
| **Algae group** | **Relative abundance in the diet (% ± SE)** | |  |
| Red calcareous algae | **48 ± 6** | 3 **±** 2 |  |
| Red corticated algae | **27 ± 6** | **81 ± 3** |  |
| Green filamentous algae | 12 ± 7 | 7 **±** 1 |  |
| Red filamentous algae | 5 ± 3 | 0 **±** 0 |  |
| Invertebrates | 6 ± 4 | 0 **±** 0 |  |
| Cyanophyceae | 2 **±** 1 | 6 **±** 2 |  |
| Green corticated algae | 0 ± 0 | 0 **±** 0 |  |
| Brown corticated algae | 0± 0 | 2 **±** 1 |  |
| Brown filamentous algae | 0 ± 0 | 1 **±** 1 |  |
